# Chromosome anchoring in Senegalese sole (*Solea senegalensis*) reveals sex-associated markers and genome rearrangements in flatfish

**DOI:** 10.1038/s41598-021-92601-5

**Published:** 2021-06-29

**Authors:** Israel Guerrero-Cózar, Jessica Gomez-Garrido, Concha Berbel, Juan F. Martinez-Blanch, Tyler Alioto, M. Gonzalo Claros, Pierre-Alexandre Gagnaire, Manuel Manchado

**Affiliations:** 1 IFAPA Centro El Toruño, Junta de Andalucía, Camino Tiro Pichón s/n, 11500 El Puerto de Santa María, Cádiz, Spain; 2 CNAG-CRG, Centre for Genomic Regulation (CRG), Barcelona Institute of Science and Technology (BIST), 08028 Barcelona, Spain; 3 Biopolis S.L.-ADM, Parc Cientific Universidad De Valencia, Edif. 2, C/ Catedrático Agustín Escardino Benlloch, 9, 46980 Paterna, Spain; 4 Universitat Pompeu Fabra (UPF), 08003 Barcelona, Spain; 5 Department of Molecular Biology and Biochemistry, Universidad de Málaga, 29071 Málaga, Spain; 6 CIBER de Enfermedades Raras (CIBERER), 29071 Málaga, Spain; 7 Institute of Biomedical Research in Málaga (IBIMA), IBIMA-RARE, 29010 Málaga, Spain; 8 Instituto de Hortofruticultura Subtropical Y Mediterránea (IHSM-UMA-CSIC), 29010 Málaga, Spain; 9 ISEM, Univ Montpellier, CNRS, EPHE, IRD, Montpellier, France; 10 Crecimiento Azul, Centro IFAPA El Toruño, Unidad Asociada al CSIC, El Puerto de Santa María, Spain

**Keywords:** Animal breeding, Genomics

## Abstract

The integration of physical and high-density genetic maps is a very useful approach to achieve chromosome-level genome assemblies. Here, the genome of a male Senegalese sole (*Solea senegalensis*) was de novo assembled and the contigs were anchored to a high-quality genetic map for chromosome-level scaffolding. Hybrid assembled genome was 609.3 Mb long and contained 3403 contigs with a N50 of 513 kb. The linkage map was constructed using 16,287 informative SNPs derived from ddRAD sequencing in 327 sole individuals from five families. Markers were assigned to 21 linkage groups with an average number of 21.9 markers per megabase. The anchoring of the physical to the genetic map positioned 1563 contigs into 21 pseudo-chromosomes covering 548.6 Mb. Comparison of genetic and physical distances indicated that the average genome-wide recombination rate was 0.23 cM/Mb and the female-to-male ratio 1.49 (female map length: 2,698.4 cM, male: 2,036.6 cM). Genomic recombination landscapes were different between sexes with crossovers mainly concentrated toward the telomeres in males while they were more uniformly distributed in females. A GWAS analysis using seven families identified 30 significant sex-associated SNP markers located in linkage group 18. The follicle-stimulating hormone receptor appeared as the most promising locus associated with sex within a region with very low recombination rates. An incomplete penetrance of sex markers with males as the heterogametic sex was determined. An interspecific comparison with other Pleuronectiformes genomes identified a high sequence similarity between homologous chromosomes, and several chromosomal rearrangements including a lineage-specific Robertsonian fusion in *S. senegalensis.*

## Introduction

Genetic maps represent essential tools for genomic research in aquaculture. Originally, linkage mapping studies were mainly based on microsatellite (SSR) and AFLP markers^1,2^; nevertheless, they recently reached a milestone with the development of genotyping methods based on cost-effective massive parallel sequencing. The genomic revolution has made single-nucleotide polymorphisms (SNPs) very popular, opening up access to a simple biallelic marker with a wide distribution and high abundance across the genome. As consequence, an increasing number of high-density genetic maps is nowadays reported in non-model organisms including aquaculture fish^3,4^. These maps have proven to be useful to provide new clues on genome evolution and speciation between closely related lineages, and to unravel the genetic architecture of both simple Mendelian and complex quantitative traits in many fish species, thus facilitating marker-assisted selection in aquaculture^5,6^. More recently, a new application of high-density linkage maps as backbones to anchor de novo genome assemblies into pseudo-chromosomes has become more widespread^7,8^. Although long-read sequences have significantly enhanced the average size of scaffolds in de novo assembled genomes^9^, the total number of scaffolds are still far beyond the expected number of chromosomes. The large arrays of repeated sequences and the degree of conservation for some tandem repeats families widely distributed across the genome still remain a major obstacle for most de novo assembly algorithms, resulting in fragmented scaffolds or even misassembled sequences within chimeric contigs. Linkage maps thus provide highly valuable tools to anchor physical maps into pseudo-chromosomes, while enabling the identification of chimeric or misassembled contigs towards enhancing the quality of new genome assemblies^7^.

Flatfish (Pleuronectiformes) is an attractive group of fish that have long been investigated due to the drastic morphological, physiological and behavioural remodelling changes that occur during metamorphosis from a pelagic larva to a benthic juvenile stage. Several flatfish species are worldwide exploited in fisheries and aquaculture, thus representing an important resource for human consumption. This taxonomic group diverged from carangimorphs in the early Paleocene, and underwent a major diversification in the middle Paleocene^10^. Cytogenetic studies have suggested that the Pleuronectiformes ancestor should have 2n = 48 chromosomes in agreement with the most frequent number of chromosomes found in the sister clade Carangidae, and in the most deep-branching flatfish families (Pleuronectidae and Paralichthyidae)^11^. However, the number of chromosomes in flatfish encompasses a wide range varying from 2n = 26 to 2n = 50^11,12^. An intense cascade of Robertsonian rearrangements and pericentromeric inversions seems to have shaped flatfish genome evolution, especially reducing the chromosome number in most recently diverged families of Soleidae, Cynoglossidae and Achiridae^11^. A recent comparison of the turbot genome with other fish assemblies clearly pointed out the high degree of conserved synteny across chromosomes in Pleuronectiformes, although with high rates of intrachromosomal reorganisations. Moreover, some chromosome fusions identified through comparative mapping are thought to have given arise to a new karyotype organization in turbot^3^. Hence, integrated genetic and physical maps are important genomic resources to understand chromosome evolution in flatfish.

The Senegalese sole is an important flatfish in aquaculture and fisheries. A genetic linkage map based on 129 SSRs grouped into 27 linkage groups (LG) was previously reported^13^. Moreover, an integrated map using BAC clones and repetitive DNA families was also developed using a multiple fluorescence in situ hybridization (mFISH) technique with at least one BAC mapped to each chromosome arm^14^. This cytogenetic study evidenced a lack of heteromorphic sex chromosomes and identified the largest metacentric chromosome to result from a Robertsonian fusion of two acrocentric chromosomes during flatfish evolution^15,16^. Moreover, a preliminary draft genome sequence of a female Senegalese sole was reported (600.3 Mb, N50 of 85 kb), and then further improved with a hybrid assembly using Nanopore and Illumina reads (608 Mb long, N50 of 340 kb)^17,18^. This genome information was used to design whole-genome multiplex PCR and create a new integrated SSR map with 234 markers. Nevertheless, further efforts are required to better assemble and anchor scaffolds onto the 21 expected chromosomes, and to better understand the genomic architecture of sex-determination.

The aim of this study was to: (1) generate an improved de novo assembly of a male Senegalese sole based on a combination of long and short read sequencing; (2) build a high-density genetic map using ddRAD markers; (3) anchor the physical to the genetic map in order to (4) improve the scaffolding of the reference genome assembly; (5) estimate genome-wide variation in recombination rates; and (6) carry out GWAS analysis to identify sex-associated markers and intra- and interspecific comparative mapping to better understand the evolutionary history of chromosome rearrangements in flatfish.

## Material and methods

### Animals

Soles used for the preparation of ddRAD libraries and sequencing were selected from the genetic breeding program carried out by the IFAPA in collaboration with a commercial aquaculture company (CUPIMAR S.A.). Production of families used in this study, genotyping and parentage assignment were previously published^19,20^. Five families (three full-sib and two maternal half-sib families) containing between 48 and 96 individuals per family (total n = 356) were selected to construct the genetic linkage map (Table 1). Moreover, seven families with sex ratios close to 1:1 were selected for genome-wide association analysis (GWAS). Average weight and length of each family are depicted in Table 1. As genotyping of parents was also required to build the genetic map, five fathers and three mothers involved in family production were sampled for blood by puncturing in the caudal vein using a heparinized syringe, adding heparin (100 mU) and keeping at − 20 °C until use. To obtain high-molecular weight genomic DNA for genome sequencing, a wild male from the broodstock (weight higher than 2 kg; code Sse05_10M) was sampled for blood as indicated above.

**Table 1:** Families used to construct the genetic linkage map (LM) and association study (A).

Family name	Use	Parents	Weight	Length	n	nQ	Final
Fam1	LM/A	F1/M1	161.6 ± 94.3	20.6 ± 4.0	76	76	73
Fam2	LM	F2/M2	244.5 ± 157.8	22.7 ± 4.4	95	95	90
Fam3	A	F3/M3	219.3 ± 95.9	22.4 ± 3.5	68	67	65
Fam4	A	F4/M4	460.8 ± 195.4	27.8 ± 4.1	99	79	77
Fam5	LM/A	F5/M5	216.2 ± 67.1	22.5 ± 2.3	48	48	47
Fam6	LM/A	F6/M5	345.5 ± 136.2	25.6 ± 3.4	71	65	63
Fam7	LM/A	F7/M2	540.4 ± 211.3	28.6 ± 3.6	66	62	54
Fam8	A	F8/M1	129.8 ± 72.7	19.5 ± 3.9	76	73	73
TotalLM					356	346	327
TotalA					504	470	452

Father (F) and Mother (M) of each family, the average weight and standard length at age 800 days and the number of specimens originally selected for analysis (n) are indicated. Moreover, the number of animals that passed that DNA quality analysis (nQ) and the final number of animals that passed after checking for Mendelian errors.

All procedures were authorized by the Bioethics and Animal Welfare Committee of IFAPA and given the registration number 10/06/2016/101 by the National authorities for regulation of animal care and experimentation. The study was carried out in compliance with the ARRIVE guidelines and all procedures were performed in accordance with Spanish national (RD 53/2013) and European Union legislation for animal care and experimentation (Directive 86\609\EU).

### Genome sequencing and assembly

Methods for genome sequencing and assembly are fully described in “Supplementary method”. Briefly, high-molecular weight genomic DNA was prepared from heparinized whole blood using the MagAttract HMW DNA kit (Qiagen). Once confirmed quality, four libraries were prepared for sequencing using the Oxford nanopore Technology (ONT) MinION platform. Overall, 19.2 Gb of genome information was generated with an average read length of 4.3 kb. In parallel, the same sample was also sequenced in a NextSeq550 sequencer (Illumina, USA) that overall generated 43 Gb of sequence from 143 million reads (average length 147 nt). The main features of the libraries used during the genome assembly are presented in Supplementary Table S1. The raw read data were deposited to the NCBI Sequence Read Archive (SRA) under accession number SAMN16809702. The hybrid genome assembly was carried out using MaSuRCA*v*3.2.3^21,22^ with the Illumina libraries (57.3 × coverage) and the error-corrected Nanopore reads (25.5x). The LR-hybrid assembly was characterized for completeness using Benchmarking Universal Single-Copy Orthologs (BUSCO*v*3.0.2)^23,24^ containing 4,854 single-copy orthologs from actinopterygii_odb9.

### ddRAD-seq library preparation and sequencing

Genomic DNA from the caudal fin (offspring) or whole blood (parents) were purified using the Isolate II Genomic DNA Kit (Bioline). DNA was sent to the company LifeSequencing S.L. (Valencia, Spain) and a total of 346 samples were selected for library construction (Table 1). Libraries were constructed based on the protocol described by Peterson et al.^25^ using the EcoRI/NcoI enzyme combination that generated as average 24,874 SNPs per sample. Pools of libraries were loaded on a Novaseq 6000 sequencer (Illumina), following the manufacturer's instructions and the specifications mentioned above. The total number of reads generated for each library are indicated in Supplementary Table S2.

### Genetic linkage map and scaffold anchoring

**Figure 1:**
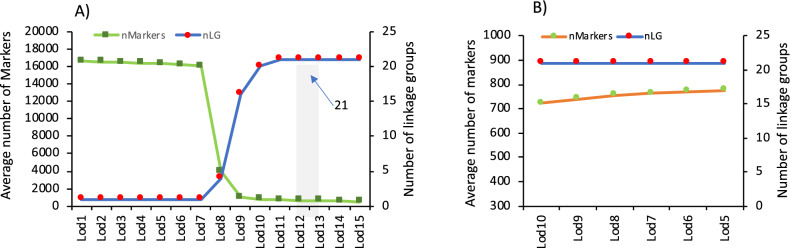
Selection of LOD score limit (Lod) to construct genetic map in LepMap3. (**A**) The average of number of markers (nMarkers) positioned in linkage groups (left Y axis) and the number of linkage groups (nLG; right Y axis) for Lod values from 1 to 15 as implemented in the "SeparateChromosomes” module. Lod11 (shaded) indicates the value selected that grouped the markers in 21 LGs. (**B**) Average number of markers recovered and added to the 21 LGs using decreasing LOD score iterations from 10 to 5 in the JoinSingles2 module.

Illumina reads were processed using Stacks v2.3e^26^ as indicated in “Supplementary method”. To construct the map, SNPs were filtered using Plink v1.9^27^ to remove markers that segregated with Mendelian errors in more than 10% of individuals. Moreover, those individuals with more than 5% of markers with Mendelian errors were removed (Supplementary Fig. S1). The final SNP dataset contained 40,041 markers from 327 individuals (Table 1) and 8 parents that were imported in LepMap3^7^. The SNPs were assigned to 21 linkage groups (named as SseLGs) corresponding to the expected number of chromosomes (2n = 42) using the·"SeparateChromosomes” module. A LOD threshold of 11 and a size limit of 200 were selected as the most adequate parameters to keep an optimal number of markers grouped in the expected number of SseLGs (Fig. 1A,B). Module JoinSingles2 was run to assign additional single SNPs to existing SseLG using decreasing LOD score iterations from 10 to 5 (Fig. 1B). Finally, the genetic distances between markers on each SseLG was calculated with the OrderMarkers2 module (male, female, sex average (SA)) using the Kosambi mapping function. The resulting genetic map was visualized using the software linkagemapview^28^. Scaffolds anchoring was carried out using the Lep-Anchor program following the author's recommendation^29^ and indicated in “Supplementary method”.

### Genome annotation

Genome annotation was performed by combining alignments of *Danio rerio*, *S. maximus* and *S. semilaevis* proteins, RNAseq from several tissues and developmental stages alignments and ab initio gene predictions. Annotation process is described in “Supplementary method” with a higher detail. Functional annotation was performed on the male annotated proteins with Blast2GO^30^. After performing an alignment-based strategy to determine equivalences between female and male genomes (see “Supplementary method”), the female proteins inherited the functional annotation of their male equivalences. Next, functional annotation was performed in the female genes that remained unannotated after this step. Gene Ontology (GO) enrichment was carried out with topGO in those genes that were unique to one of the genomes (Supplementary Table S3).

### Recombination rates, association analyses and cross-species comparisons

Recombination rate variation along the genome was evaluated by comparing the consensus linkage map for both sexes and SA and the physical map of each pseudo-chromosome using MareyMap^31^. The cumulative recombination frequency (RFm) along LGs was used to infer the chromosome type as previously described^32^. GWAS analysis were carried out with seven families (Table 1) using a logistic mixed model (multi-step) approach as implemented in the R package GENABEL (v1.8–0)^33^ for binary traits (Female = 0 and Male = 1). A highly detailed analysis of synteny across flatfish is beyond the scope of this study, but a chromosome alignment analysis was carried out to identify chromosomal rearrangements in flatfish using D-Genies^34^. We then used the SatsumaSynteny to compute whole-genome synteny blocks^35^ that were later represented using Shinycircos^36^.

## Results

### Male genome assembly and annotation

**Figure 2:**
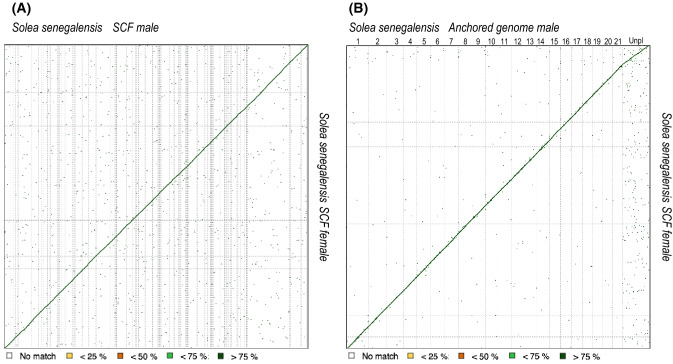
Dot plot comparison of scaffolds (SCF) assembled (**A**) or 21 pseudo-chromosomes (**B**) in the male with respect to SCF in the female. Scale is indicated below.

A de novo hybrid genome for a male sole was assembled using a combination of Illumina and Nanopore long-reads. Main features about the total number of input reads used for each sequencing platform, the average read length and quality and total sequencing information used in the assembly are indicated in Supplementary Table S1. The hybrid assembly draft sequence was generated using MaSuRCA and later refined with Pilon to correct bases, mis-assemblies and filling gaps. Main statistics about the assembly are depicted in Supplementary Table S4. The new assembly consists of 3,403 contigs with a total length of 609,359,514 bp, and a N50 of 513 kb. Overall, 49.4% of contigs had a size longer than 50 kb and the largest fragment was 4.5 Mb long. The estimated gene integrity, as determined by BUSCO analysis, revealed 97.0% completeness. For comparison purposes, the assembly statistics for a recent female genome draft of *S. senegalensis*^20,20^ are also shown in Supplementary Table S4. Both genome assemblies had a similar size (608–610 Mb) although the newly assembled male genome had longer contigs with higher N50 values. A dot-plot alignment using the scaffolds of both genomes indicated that with 92.8% of genomic information highly similar (> 75%) and only 5.3% had no similarity (average similarity 94%) (Fig. 2).

Assembly annotation statistics are depicted in Table 2. The number of protein-coding genes in the male assembly (27,175) was slightly lower than in the female (28,988) but with a longer mean length (7.4 vs 6.7 kb). The estimated percentages of annotated transcripts (69.4–72.1%) and gene density (45.03–47.68) were similar between both assemblies. Around 85% of the annotated genes in each assembly had an equivalent gene in the other assembly. However, a few genes were only present in one of the genomes (unique genes). Some of these gene differences might be due to genome heterozygosity and repeat content or even sex-specific genes. A GO enrichment analysis using these unique genes indicated that categories related to the cell-cycle regulation and regulation of transcription, involving canonical histones H3.2 and H4 and retinoid X receptor alpha (*rxra*), were highly significantly overrepresented in the female (p-value < 10^–3^). Mapping of these two histone genes on female assembly showed that they were co-localized in five scaffolds (Sosen1_s0284, Sosen1_s0324, Sosen1_s1454, Sosen1_s1522, Sosen1_s1726), four of which clustered in SseLG1 and one in SseLG16. In male, the most significant enriched categories for unique genes were skeletal system development and morphogenesis although with *P*-values > 0.001 (Supplementary Table S3). Some short, single-exonic unique genes might be the result of scaffold splitting or annotation processes. The non-coding gene annotation resulted in 23,822 female and 21,123 male transcripts, respectively. From these, 6,549 and 6,007 female and male transcripts were long non-coding RNAs (lncRNAs) and the rest short non-coding RNAs.

**Table 2:** Summary annotation statistics for male and female assemblies.

	Male	Female^#^
Repeat content	23.55%	23.41%
Number of protein-coding genes	27,175	28,988
Median gene length (bp)	7,368	6,721
Number of transcripts	50,133	51,844
Number of exons	303,132	307,753
Number of coding exons	284,414	288,788
Coding GC content	52.67%	52.57%
Median UTR length (bp)	1,231	1,222
Median intron length (bp)	388	371
Exons/transcript	11.88	11,53
Transcripts/gene	1.84	1.79
Multi-exonic transcripts	0.956	0.941
Gene density (gene/Mb)	45.026	47.679
Functionally annotated transcripts	36,130 (72.1%)	35,999 (69.4%)
Unique genes	3,806 (14%)	4,643 (16%)
non-conding RNAs	21,123	23,822

Annotation pipeline is described with more details in “Supplementary method”.

^#^Sequence deposited in figshare https://doi.org/10.6084/m9.figshare.12472100.v1.

### ddRAD sequencing and SNP detection for genetic linkage map

Three full-sib and two half-sib families consisting of 47 to 95 individuals were used for ddRAD analysis (Table 1). The total number of paired-end reads generated for each family ranged between 280,609,738 (F5) and 398,313,256 (F2) with an average length of 150 nt (Table 3). The average number of reads per individual in each family varied between 6,444,752 (F1) and 11,692,072 (F5) (Table 3 and Supplementary Table S2). For parents, the average number of reads was 8,847,913.

**Table 3:** Main statistics of ddRAD libraries, mapping and SNP detection.

	n	Total reads family	Av. raw reads	Av. reads stacks	PA (%)	Unmapped	loci	mean cov	n_gts
F1	76	244,900,564	6,444,752	6,215,911	88.23	0.34%	23,828	146	22,040
F2	95	398,313,256	8,385,542	8,090,267	89.71	0.33%	24,978	190	22,823
F3	67	226,072,540	6,649,192	6,090,258	86.20	0.32%	26,068	132	24,054
F4	79	248,271,546	6,130,162	5,972,512	87.74	0.33%	25,525	135	23,157
F5	48	280,609,738	11,692,072	11,384,985	88.13	0.33%	30,005	237	27,011
F6	65	363,499,961	11,184,614	10,899,007	88.04	0.31%	27,742	242	24,883
F7	62	337,573,225	10,889,459	10,627,007	88.93	0.34%	30,550	226	26,773
F8	73	447,768,745	12,267,637	11,674,383	89.42	0.33%	28,002	260	25,371
Parents	8	39,815,609	8,847,913	8,323,338	86.08	0.36%	17,632	242	15,898

The total number of individuals analysed (n), the total reads per family, the average number of paired-end reads per individual, the average number reads used by stacks, the % of primary alignment and unmapped reads, number of *loci*, effective coverage, and number of genotypes (n_gts).

The new assembled male genome was used as reference to map the ddRAD reads. The average fraction of primary alignments onto this reference genome ranged between 88.04 (F6) and 89.71% (F2). An average of 10.5% of reads had insufficient mapping qualities or excessively soft-clipped primary alignments while less than 0.34% were unmapped. A total of 199,188 ddRAD *loci* were reconstructed with an average number of loci per sample ranging between 23,828 (F1) and 30,550 (F7) and a mean insert length of 330.7 bp. The effective coverage per sample was 193.3 ± 110.4 (ranging from 146 to 242 between families) and the estimated mean number of sites per locus was 242.8 (Table 3).

### Construction of a linkage genetic map and anchoring to physical map

To construct the genetic map, only those SNPs detectable in at least 80% of samples with a coverage of 10 reads per sample were considered. Moreover, SNPs with a significant deviation from Mendelian segregation were also removed (a total of 2,439 markers, 5.7% SNPs). By family, the number of markers with Mendelian errors ranged from 1.5 to 1.7% (Supplementary Fig. S1). Moreover, those animals with markers that had more than 5% of Mendelian errors (19 specimens) were also removed. Overall, the final dataset contained 40,041 SNPs segregating in eight parents and their 327 offspring.

**Figure 3:**
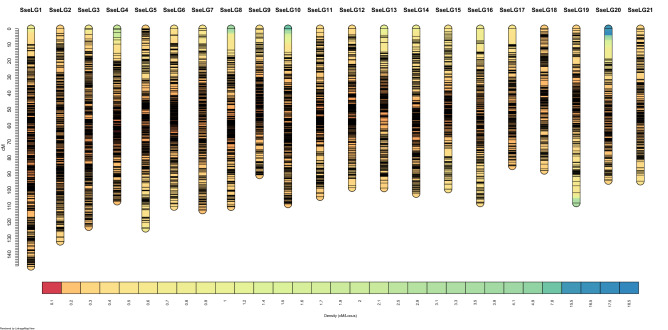
Genetic distance (cM) and SNP distribution across 21 linkage groups (SseLG) of the Senegalese sole.

For linkage analysis, the ParentCall2 module retained only 16,287 informative markers after checking for segregation distortion (*P* < 0.05). Markers grouped into 21 SseLGs (via the SeparateChromosomes2 module) with a LOD = 11 (Fig. 1), which is consistent with the number of chromosomes in *S. senegalensis*. Each SseLG contained between 530 and 1,337 markers with an average number of 21.9 markers per Mb (Fig. 3, Table 4 "Anchoring genetic map and physical map"). In total, the genetic map allowed the anchoring and positioning of 1,665 out of 3,403 total contigs, ranging between 50 to 129 contigs in each SseLG. The genome sequence positioned on the linkage map was larger (746.3 bp) than the assembly size, mainly due to the presence of chimeric contigs (n = 133) positioned in various chromosomes.

**Table 4:** Information for anchored physical map (LepMap3 step), after genome re-scaffolding (Lep-anchor3 step) and after removal of markers with discrepancies between genetic and physical maps (MareyMap step).

	Anchoring genetic map and physical map					Genome re-scaffolding						Marker refining		
	**Length (bp)**	**nMar**	**nCont**	**ACL**	**M/Mb**	**Length(bp)**	**NM**	**nCont**	**ACL**	**L(cM)**	**M/Mb**	**NMar**	**Mb/cM**	**M/Mb**
**1**	59,220,137	1,337	129	459,071	22.6	42,924,012	1,323	124	343,392	147.3	30.8	1,296	0.29	30.2
**2**	42,658,310	1,054	91	468,773	24.7	36,396,255	1,046	88	413,594	131.8	28.7	1,032	0.28	28.4
**3**	47,587,809	1,015	85	559,857	21.3	33,319,822	1,006	80	416,498	136.6	30.2	978	0.24	29.4
**4**	42,630,187	920	83	513,617	21.6	27,129,084	899	73	366,609	106.9	33.1	885	0.25	32.6
**5**	32,366,427	891	86	376,354	27.5	27,692,037	872	78	350,532	142.5	31.5	811	0.19	29.3
**6**	34,539,569	864	80	431,745	25.0	26,866,643	860	77	348,917	114.0	32.0	832	0.24	31.0
**7**	36,891,773	849	87	424,043	23.0	28,334,760	836	77	367,984	133.8	29.5	795	0.21	28.1
**8**	36,615,909	784	86	425,766	21.4	27,361,452	769	82	333,676	119.3	28.1	756	0.23	27.6
**9**	32,328,246	804	65	497,358	24.9	25,679,769	802	63	407,615	105.1	31.2	765	0.24	29.8
**10**	35,518,751	768	88	403,622	21.6	25,170,845	762	84	299,653	113.7	30.3	748	0.22	29.7
**11**	37,595,336	780	99	379,751	20.7	26,846,769	769	93	288,675	126.2	28.6	732	0.21	27.3
**12**	37,197,923	763	80	464,974	20.5	25,840,656	752	77	335,593	98.5	29.1	731	0.26	28.3
**13**	34,656,556	665	50	693,131	19.2	23,154,965	658	48	482,395	98.7	28.4	637	0.24	27.5
**14**	33,597,656	668	76	442,074	19.9	26,091,242	665	74	352,584	109.5	25.5	637	0.24	24.4
**15**	36,416,189	644	66	551,760	17.7	22,903,974	632	59	388,203	113.1	27.6	601	0.20	26.2
**16**	26,721,177	630	58	460,710	23.6	21,637,702	618	52	416,110	108.0	28.6	602	0.20	27.8
**17**	30,251,165	616	79	382,926	20.4	21,095,432	610	75	277,572	103.3	28.9	563	0.20	26.7
**18**	24,300,965	587	62	391,951	24.2	19,718,726	577	57	345,943	87.8	29.3	561	0.23	28.5
**19**	36,478,108	584	75	486,375	16.0	21,051,312	575	70	296,497	108.0	27.3	562	0.20	26.7
**20**	24,034,263	534	62	387,649	22.2	20,166,255	530	62	325,262	105.7	26.3	497	0.19	24.6
**21**	24,720,343	530	78	316,928	21.4	19,202,697	514	70	270,461	98.3	26.8	490	0.20	25.5
**ST**	746,326,799	16,287	1,665	453,259	21.9	548,584,409	16,075	1,563	349,640	2,408.1	29.3	15,511	0.23	28.3
Not-anchored			1,738			61,859,804	212	1,840				776		
**Total**	746,326,799	16,287	3,403	453,259	21.9	610,444,213	16,287	3,403				16,287		

The physical (bp) and genetic (cM) length of each linkage group, number of markers (nMar), number of contigs (nCon), average contig length (ACL), marker density density (markers per megabase; M/Mb) and the ratio physical to genetic length (Mb/cM) for sex-average genetic-physical map are indicated.

### Rescaffolding of reference genome with the genetic map

SNP marker information was further used for fine-scale correction of genome contigs to build 21 pseudo-chromosomes. After masking the repetitive sequences, the contigs were orientated and sorted within each SseLG (Table 4 "Genome re-scaffolding"). The total number of positioned contigs reduced from 1,665 to 1,563. Lep-anchor corrected the contig errors removing six contigs, splitting another 105 into two fragments, 20 in three fragments, and two in more than four fragments. After these corrections, the total number of markers assigned to the SseLGs decreased by 1.3% (16,075 SNPs) and 212 markers were moved to unplaced with an average density of 10.3 markers per contig. After these corrections, 548.6 Mb out of the 610.4 Mb total assembly length (89.9%) were assigned to the 21 SseLGs and only 61.9 Mb remained as unanchored (Table 4). The total map length was 2,408.1 cM, SseLG1 was the largest group (42,924,012 bp and 147.3 cM) and SseLG4 showed the highest marker density per megabase (33.1). The average marker interval reached 0.155 cM. A further refining of anchored markers was carried out through the comparison of physical and genetic distance in MareyMap. The average genome-wide recombination rate (RR) was 4.35 cM/Mb (ranging between 3.45 and 5.26 cM/Mb among chromosomes) (Table 4 "Marker refining"). An alignment of the anchored and refined reference male genome with the scaffolds of the female assembly (Fig. 2B) slightly increased to 93.2% the regions with more than 75% similarity and provided a clear sequence alignment in the diagonal with only dispersion in unplaced scaffolds.

### Analysis of recombination rates

**Figure 4:**
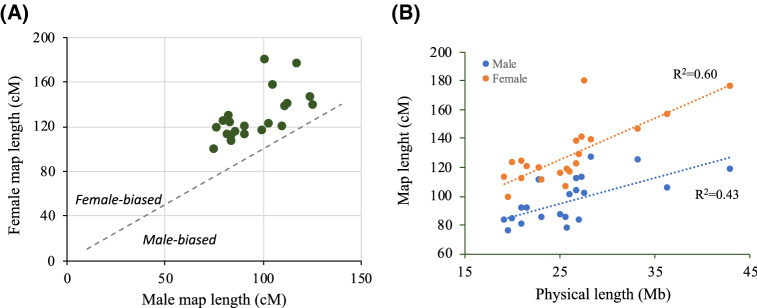
Comparison of male and female genetic maps. (**A**) Male *vs* female linkage groups lengths (cM) for the 21 Senegalese sole chromosomes. All chromosomes exhibit female-biased recombination. (**B**) Correlation between recombination map and physical map lengths in both males (blue) and females (orange). The determination coefficient R^2^ is shown separately for each sex.

Consensus genetic maps for female and male were 2,698.4 cM (15,022 markers) and 2,036.6 cM (15,390 markers), respectively. These differences in map size were observable for the 21 SseLGs (Fig. 4A and Table 5). Overall, the female-to-male ratio (F:M) for genetic distances was 1.32, ranging from 1.08 (SseLG15) to 1.77 (SseLG5) (Table 5). The genetic map length of chromosomes was highly positively correlated with their physical length in both males (r = 0.43) and females (r = 0.60) (Fig. 4B). The average genome-wide RR was estimated 3.02 ± 0.37 cM/Mb in males and 4.51 ± 0.57 cM/Mb in females (Table 5). The overall female-to-male ratio (F: M) for RR was 1.49, ranging from 1.43 to 1.90 across chromosomes. In the case of males, SseLG12 showed the lowest (2.47 cM/Mb) and SseLG16 the highest (3.60) mean RR values. In females, SseLG4 had the lowest (3.57 cM/Mb) and SseLG5 the highest (5.65 cM/Mb) mean RR values.

**Table 5:** Refined genetic maps for male (M) and female (F).

	Male genetic map				Female genetic map				F:M (cM)	MRR	FRR	F/M (RR)
	nMar	L(cM)	Mb/cM	M/Mb	nMar	Length (cM)	Mb/cM	M/Mb				
**1**	1,297	117.7	0.37	30.2	1,254	175.7	0.24	29.2	1.49	2.56	4.05	1.58
**2**	1,027	105.5	0.35	28.2	998	156.2	0.23	27.4	1.48	2.64	4.09	1.55
**3**	976	124.9	0.27	29.3	962	145.9	0.23	28.9	1.17	3.05	4.15	1.36
**4**	881	83.3	0.33	32.5	868	128.8	0.21	32.0	1.55	2.58	3.57	1.38
**5**	811	101.4	0.27	29.3	811	179.5	0.15	29.3	1.77	3.38	5.65	1.67
**6**	833	103.6	0.26	31	814	122.2	0.22	30.3	1.18	3.24	4.50	1.39
**7**	786	126.3	0.22	27.7	777	138.2	0.21	27.4	1.09	2.73	4.75	1.74
**8**	737	112.9	0.24	26.9	758	140	0.20	27.7	1.24	3.15	3.94	1.25
**9**	757	84.8	0.30	29.5	762	106.4	0.24	29.7	1.25	2.78	4.12	1.48
**10**	732	86.6	0.29	29.1	713	115	0.22	28.3	1.33	3.50	4.50	1.28
**11**	722	111.8	0.24	26.9	724	137.6	0.20	27.0	1.23	3.16	3.85	1.22
**12**	709	77.3	0.33	27.4	677	118.2	0.22	26.2	1.53	2.47	4.70	1.90
**13**	628	84.6	0.27	27.1	613	110.7	0.21	26.5	1.31	2.76	4.15	1.50
**14**	645	100.3	0.26	24.7	608	116.4	0.22	23.3	1.16	2.99	4.10	1.37
**15**	609	110.5	0.21	26.6	574	119.3	0.19	25.1	1.08	2.64	4.41	1.67
**16**	575	91.6	0.24	26.6	580	119.6	0.18	26.8	1.31	3.60	5.15	1.43
**17**	585	80.1	0.26	27.7	540	123.7	0.17	25.6	1.54	3.38	5.17	1.53
**18**	552	75.4	0.26	28	542	98.5	0.20	27.5	1.31	3.05	4.87	1.60
**19**	555	91.2	0.23	26.4	543	111.8	0.19	25.8	1.23	3.58	5.33	1.49
**20**	502	84.1	0.24	24.9	458	122.7	0.16	22.7	1.46	2.64	4.26	1.61
**21**	471	82.7	0.23	24.5	446	112.1	0.17	23.2	1.36	3.47	5.38	1.55
**ST**	15,390	2,036.6	0.27	28.1	15,022	2,698.4	0.20	27.4	1.32	3.02	4.51	1.49
**NA**	897				1,265							
**Total**	16,287				16,287							

The genetic (cM) length of each linkage group, number of markers (nMar), the ratio physical to genetic length (Mb/cM), marker density (markers per megabase; M/Mb), the F:M ratio of genetic map length, the recombination rates (RR) in both sexes and the F:M ratio of RR are indicated.

**Figure 5:**
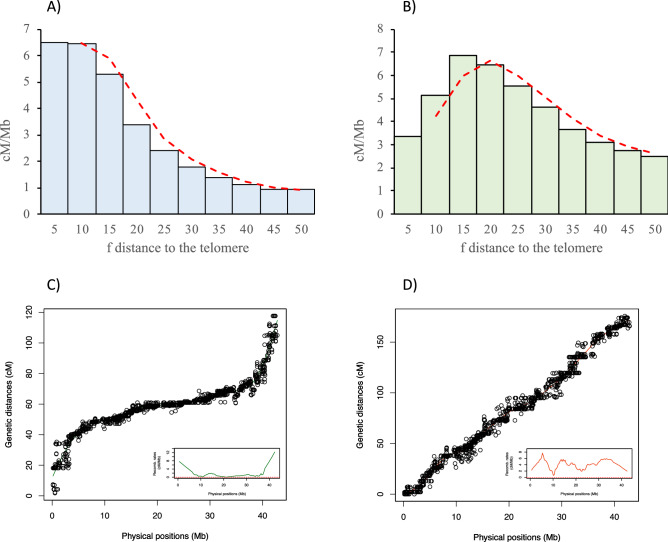
Recombination landscape averaged across linkage groups for (**A**) male and (**B**) female. The recombination rates (cM/Mb) and the relative distance from the nearest telomere scaled by the chromosome length (f) is represented. The red dashed line indicates the observed tendency. Panels (**C**,**D**) show the relationship between physical and genetic distances for SseLG1 in male and female, respectively. The square inside the panels (**C**,**D**) show the specific recombination landscape. The complete information for all SseLGs is shown in Supplementary Fig. S2 and S3.

**Figure 6:**
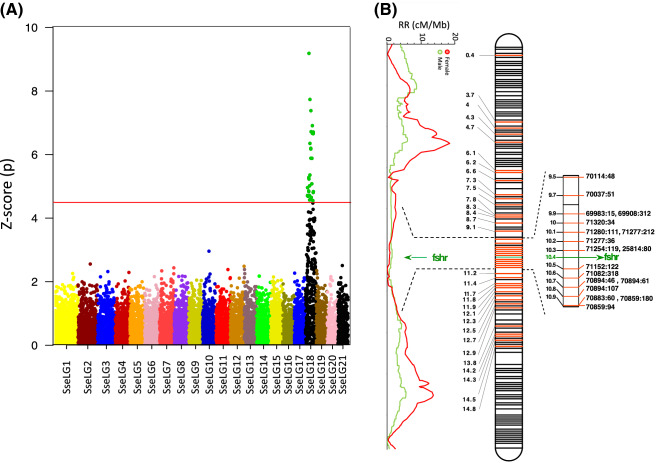
Sex-associated SNPs and RR landscape for males and females in SseLG18. (**A**) Manhattan plot of GWAS results for sex-associated SNPs using seven families. Significant markers are indicated in green. The horizontal red line represents the Bonferroni significance threshold. (**B**) Distribution of all 66 sex-associated significant markers using seven families and by family (in red, Supplementary Table S5) and RR (cM/Mb) landscape of males and females. A hot region from 9.5 to 10.9 Mb containing the candidate gene *fshr* is indicated on the right side. Physical positions of SseLG18 in Mb are indicated in black. Black lines indicate non-significant markers in SseLG18.

**Figure 7:**
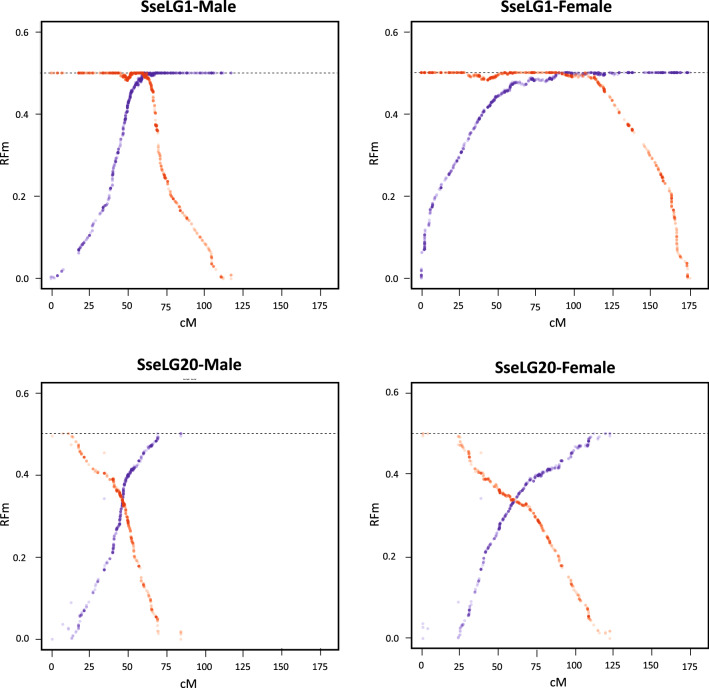
Plots illustrating the recombination frequency estimates (RFm) for intervals between markers along SseLG1 and SseLG20 in the male and female. For each LG, RFm was calculated from both chromosomal extremities (right: red circles; left: blue circles), using each of the two terminal markers as a reference starting point. The RFm plots of SseLG1 and SseLG20 show a classical metacentric and acrocentric pattern, respectively. The RFm plots of all SseLGs are illustrated in Supplementary Fig. S4.

The local RR value as estimated by the relative distance to the nearest telomere was clearly different between males and females. High RR values were mainly concentrated close to the telomeres in males (Fig. 5A), while they were more uniformly distributed in females with higher RR being found around 15% of the distance to the nearest telomere (Fig. 5B). This was illustrated by contrasted chromosomal RR landscapes between males and females, as shown Fig. 5C,D for SseLG1 (landscape for all SseLGs are represented in the Supplementary Fig. S2 for males and Supplementary Fig. S3 for females). We detected some regions within SseLGs (i.e. 5, 11, 13, 14, 15, 18) with very low RR. In the case of SsseLG18, partially restricted male or female RR was detected in the region comprised between 9.5 and 10.9 Mb. This region had very low RR in males (1.2) and females (0.6) compared with average SseLG18 (3.0 and 4.9 RR, respectively). Cumulative RR crossed between both sexes around chromosomal position 10 Mb with female RR closed to zero in 10.8–10.9 Mb (Fig. 6, Supplementary Fig. S2 and S3). Moreover, recombination frequencies were used to describe and classify chromosome morphologies. Figure 7 depicts the typical RFm plots for an acrocentric (SseLG20) and a metacentric (SseLG1) chromosome (for all SseLG see Supplementary Fig. S4).

### Association analyses for sex

To identify genome regions associated with sex, a GWAS analysis was carried using seven families (Table 1) and a total of 10 426 markers. Data for RAD-seq data and markers are indicated in Table 3. The results showed 30 markers significantly associated with sex after bonferroni correction using seven families (*P* ≤ 4.8 × 10^–6^; Fig. 6A and Supplementary Table S5). When the association analysis was repeated separately by family, five families provided some new 36 significant markers (Supplementary Table S5). All of them (66 SNPs including the whole-population and families) were spread in the SseLG18 with a hot region around 9.5–10.9 Mb (Fig. 6B). RR in this region was low (see above) with partially restricted RR associated with sex. Overall, 80.7% of significant markers using the whole population were preferentially heterozygous in males although penetrance was incomplete in most of them. This model is compatible with a nascent XY system. It should be noted that specific markers in family 4 had an expected high number of heterozygous *loci* in females.

To detect candidate sex-related genes, the full-length transcriptome^38^ was blasted onto the SseLG18 (Supplementary Fig. S5) and a total of 229 genes were positioned. The significant SNPs were highly distributed through the pseudo-chromosome, but the follicle stimulating hormone receptor (*fshr*) gene just appeared located in the hot region revealing as a clear candidate gene for sex determination.

### Interspecific chromosome rearrangements

**Figure 8:**
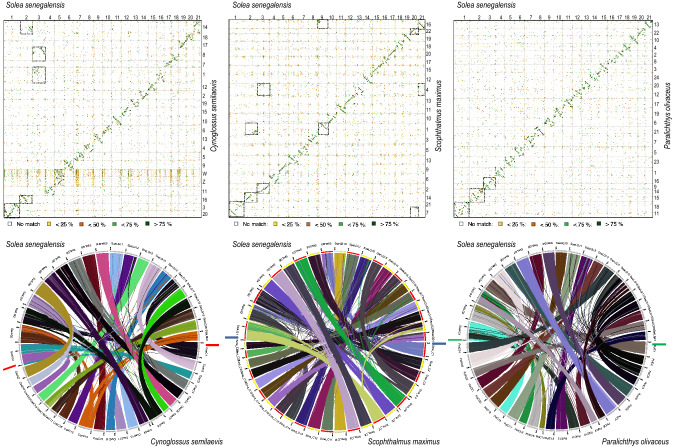
Chromosomal alignment and synteny analysis between flatfish genomes. Top panel, Dot plot comparison of 21 pseudo-chromosomes of *S. senegalensis* with the genomes of the flatfish *C. semilaevis* (left), *S. maximus* (center) and *P. olivaceus* (right). Chromosome numbers or SseLGs are indicated. The chromosome fusions are boxed. Identity scale is indicated below. Bottom panel, syntenic comparison between flatfish genomes.

An alignment of SseLGs pseudo-chromosomes with the chromosomes of three other Pleuronectiformes genomes (*Cynoglossus semilaevis*, *Scophthalmus maximus*, *Paralichthys olivaceus*) showed high similarity rates of and conserved macrosynteny level for fifteen out of 21 SseLGs (Fig. 8 and Supplementary Table S6). However, deviations from diagonal in the dot plot alignment indicated extensive intrachromosomal rearrangements among species. The three largest SseLGs appeared to be the result of total or partial chromosome fusions when compared with other flatfish genomes (Supplementary Fig. S6 and S7), and *S. maximus* seemed to be the flatfish species with the highest number of chromosome rearrangements between the four species compared. Genome comparisons using D-Genies^34^ indicated that the highest similarity was with *P. olivaceus* (no match 57.3%), followed by *S. maximus* (no match 59.6%), and *C. semilaevis* (no match 78.4%).

When the reduction of the number of chromosomes was explored three main Robertsonian fusions in the SseLG1 (Chr18-Chr11), SseLG2 (Chr14-Chr15) and SseLG3 (Chr9-Chr16) could explain the reduction from n = 24 in *P. olivaceus* to n = 21 in *S. senegalensis* (Fig. 7, Supplementary Fig. S6 and S7 and Supplementary Table S6). When compared to *S. maximus* (n = 22), the SseLG1 appeared as a fusion of Chr7 and Chr21. Moreover, translocations of regions from Chr1, Chr4, Chr7, Chr14 and Chr16 were also observed. In the case of *C semilaevis* with sexual chromosomes (ZW) and the same number of chromosome than *S. senegalensis,* a Robertsonian fusion in SseLG1 between Chr3–Chr20 was observed. Moreover, the SseLG3 appeared as a new chromosome resulting of the fission of Chr1 (mainly located in SseLG16) and Chr8 (mainly located in SseLG18). Two other major features in this species with respect to *S. senegalensis* were: (i) a translocation of a Chr14 region to Chr16 to create the SseLG2; and (ii) sexual ZW chromosomes appear concentrated in SseLG5 although high similar sequences are widely distributed throughout the genome. Comparison among all flatfish species (Fig. 7, Supplementary Fig. S6 and S7, Supplementary Table S6) indicated that those chromosomal regions associated with SseLG2 and SseLG3 were mainly involved in the changes of karyotypes of the four Pleuronectiformes species whereas the SseLG1 arose as a lineage-specific fusion event.

## Discussion

Genome assemblies and genetic linkage maps provide complementary information that can be integrated to produce high-quality physical maps. The resulting accurate chromosome assemblies are suitable to investigate genome evolution and species diversification, the genetic architecture of QTLs and the regulation of targeted genome regions. In this study, a de novo hybrid assembly for a male sole and a high-density SNP map were generated and combined to provide a polished draft assembly of 21 pseudo-chromosomes. A genome for a female sole was previously reported^17^ although it was highly fragmented (N50 = 85 kb, 600.3 MB long). Later, this assembly was improved by integrating Nanopore and Illumina reads, resulting in 5,748 contigs with N50 = 339.9 kb and 608 Mb long^20^ (Supplementary Table S4). In this study, the newly obtained male assembly has a lower number of contigs (3,403) and higher N50 (512.7 kb) and confirmed that the genome size of sole is around 609 Mb. This genome size is similar or even a bit larger than other flatfish^39–42^. A dot-plot alignment analysis indicated a high similarity between male and female genome assemblies perfected aligned along the diagonal (Fig. 2) with a completeness similar to other high-quality fish assemblies (> 95.5% complete genes)^40,43,44^.

Male genome characterization identified 50,133 transcripts and 27,175 protein-coding that agrees with the number of predicted transcripts in a recently assembled informative transcriptome^38^. Moreover, a small subset of unique genes was identified in both sexes with a high overrepresentation of cell-cycle regulation and regulation of transcription categories (including mainly the histones H3.2 and H4) in the female. In mammals, unique histone variants are specifically expressed in spermatogenic cells^45^. Moreover, expansion of histone multigene clusters in scleractinians was associated with sexually dimorphic expression of some variants playing a role in the control of gene expression in female and male germ cells during gametogenesis^46^. In sole, at least two *loci* of canonical histones in the largest metacentric chromosome SseLG1 linked to *dmrt1,* a key determination gene in other flatfish, were reported in sole^16,39,47^. This chromosome arose after a Robertsonian fusion and intense reorganization events^12^ that could have birth to new histone clusters under purifying selection^48^. Although we cannot exclude that some differences in the number of histone copies between both genomes could be attributed to individual variation, one plausible hypothesis is that some of these histone clusters could have subfunctionalizated and acquired a role in gametogenesis in a sex-specific manner. This hypothesis is supported by the identification of a *rxra*-like receptor also represented in such GO categories able to mediate the masculinizing effects of females mediated by its ligand TBT in rockfish females ^49^.

De novo assembled male genome was used as reference to map the ddRAD sequences and construct a high-density genetic map. The sole consensus map size and the number of high-quality markers used (Fig. 3; Table 4) were similar to those reported for turbot (2,622.09 cM)^6^ and flounder (3,497.29 cM)^50^ although with a higher density of markers (only 6,647 and 12,712 SNPs in turbot and flounder, respectively). Most importantly, markers were distributed into 21 SseLGs that match with the haploid karyotype (2n = 42) of the species^51^. Until now, two genetic maps with 129–229 microsatellites were reported in Senegalese sole^13,20^ Moreover, a cytogenetic map was also published although the number of BACs did not still cover all chromosomes^14,16^. This new high-density SNP map (Fig. 3) thus represents a key step forward for future genomic studies and QTL identification with respect the current information available until now in this species.

Although hybrid assemblies using long and short sequences reads reduce genome fragmentation and increase the average scaffold sizes as observed in this study, most of de novo genome assemblies still do not reach chromosome-level with the expected number of chromosomes due to, among other factors, the repetitive fraction of the genome. To get around this limitation, information of genome-wide physical maps and dense genetic linkage maps can be integrated to assign chromosomal locations to sequence contigs^52^. This anchoring can also remove assembly artifacts and position misplaced scaffolds to increase the contiguity of the assembled scaffolds. In this study, the high-density SNP genetic map was used to anchor, sort and refine the assembled contigs. Overall, 89.9% of the genome assembly could be anchored to 21 pseudo-chromosomes and a total of 102 contigs were removed or split to separate positions in SseLGs. A similar strategy was followed in turbot using 31 families that allowed for the rearrangement of 20% of the genome assembly^3^. A comparison between male and female demonstrated a high co-linearity between our physical map and female scaffolds (only 5.53% mismatch). Although 10.1% of genome information remained as unplaced, the anchored physical map is essential for gene association analysis, synteny and cross-species studies and targeted genome resequencing. Further studies will be required to accurately anchor the remaining 61.9 Mb unanchored regions to their position in the genome.

It is well-known that the genome-wide RR differs between males and females (heterochiasmy) and that the recombination landscape also varies along chromosomes. In animals and plants, females tend to have higher RR than males, which in turn result in larger map lengths^53–55^. In our study, map was longer in the female than in the male (2,698.4 *vs* 2,036.6 cM; ratio 1.32). Assessment of sex-specific RR indicated a female-biased heterochiasmy across all SseLGs, with an average RR of 3.02 in male *vs* 4.51 cM/Mb in female. Four species of Pleuronectidae also exhibited wide heterochiasmy through all chromosomes similarly to sole with some intervals of male- and female-restricted meiotic recombination^56^. However, such differences in RR between males and females are not fully conserved in flatfish when map size is considered. Female maps are larger in turbot (1.36 times) and halibut (1.07 times)^1,2,57^, this is not the case of flounder or tongue sole with slightly larger maps in males (1.03–1.09 times)^50,58,59^. *C. semilaevis* is the only flatfish known with heteromorphic sex chromosomes (ZZ/ZW) that has been described in several mammals, birds and insects as a cause for an arrest of recombination in the heterogametic sex (XY males or ZW females). This could explain a shift in the direction of heterochiasmy^53^.

In addition to such differences in overall RR between sexes, the chromosomal recombination landscapes also differed between male and female according to typical patterns. In fish, it has been shown that recombination occurs at higher frequencies near telomeres in males while the distribution is quite more uniform or elevated near centromeres in females^54^. In stickleback fish, it has been demonstrated that centromeres and telomeres have little or no effect on recombination in females, however, in males, the recombination rates are suppressed near the centromeres and hence crossovers localize mainly at the ends of long arms in acrocentric chromosomes^55^. This feature seems to be conserved in sole since RR were also more frequent toward the end of males SseLGs compared to females (Fig. 5).

Heterochiasmy is considered a major force that guides the evolution of genetic sex determination systems and speciation^56,60^. Normally, genome regions with very low RR are associated with sex-determining regions in young sex chromosome systems and sex-linked traits such as pigmentation^61^. In Atlantic halibut, the sex determining gene *gsdf* is located in a region of chromosome 13 with restricted male and female RR^56^. In *S. senegalensis*, 30 significant sex-associated SNPs (66 if we consider the SNPs of separated families) were distributed throughout the SseLG18 with very low RR hot region (Fig. 6 and Supplementary Fig. S2 and S3). The shift and crossing between male and female RR suggest sex-specific restricted meiotic recombination events and that heterochiasmy might be involved in nascent sex chromosome system.

Most of SNP markers in the whole-population were heterozygous in males suggesting an XX/XY system. However, it should be noted high levels of incomplete penetrance in the families analysed (Supplementary Table S5). The fact that this proportion was even inverted in specific markers of F4 indicates a high effect of environmental factors on sex determination. The temperature seems to be a major factor that modifies sex ratios during larval development generating skewed populations of neomales and neofemales^62,63^. Familial sex ratios in sole were reported to oscillate from 16 up to 90% males supporting a high impact of environmental factors to modulate sex differentiation and sex population ratios^19^.

After analyzing the hot region in SseLG18, the *fshr* appeared as a putative candidate for sex determination. The *fshr* locus was recently associated with male sex in flatfhead grey mullet with an incomplete penetrance as observed in sole^64^. These authors proposed that *fshr* might act as a proxy for the genetic transduction of environmental factors such as temperature Under this hypothesis, sex determination would not rely on a single genetic cascade but a continuum of environmental and genetic factors. In sole, *fshr* was mainly expressed in testis^65^. The Fshr together with StAR are expressed in the steroidogenic Leydig cells and Fshr act as a promiscuous receptor that mediates the steroidogenic activity induced by both FSH and LH^66,67^. This double action supports a prolonged spermatogenesis and spermatid availability within the testis throughout the year mediated by FSH and the differentiation of spermatids into spermatozoa and subsequent spermiation mediated by LH^66^. Functional studies are needed to validate this putative candidate.

A synteny comparison of SseLGs with different flatfish genomes indicated that there was a one-to-one correspondence for 15 chromosomes, with some lineage-specific rearrangements (Fig. 8 and Supplementary Table S6). This correspondence between chromosomes was also confirmed when genome of turbot was compared with other flatfish demonstrating intrachromosomal rearrangements that shaped chromosome synteny and gene organization^3^. In our data, deviations from diagonal unlike in the comparison between male and female are indicative of this intense internal reorganization across species. The three SseLGs (SseLG1, SseLG2 and SseLG3) deserve special attention as they can provide an evolutionary framework to understand the history of chromosome fusions and fissions that shaped the karyotypes in flatfish. The SseLG1, predicted as a metacentric chromosome by the analysis of recombination frequency (Fig. 6), was previously identified by cross-species genomic comparison as the largest metacentric chromosome in Senegalese sole suggesting it may be a proto-sexual chromosome^12,16^. Our data support the hypothesis that this chromosome has primarily emerged by a lineage-specific Robertsonian fusion, since the homologs in other flatfish maintained their integrity across evolution (Supplementary Fig. S7). A complex series of events including small chromosomal translocations and rearrangements, fusions, and pericentric inversions would explain the current gene content and organization^12^. Unlike SseLG1, the SseLG2 and SseLG3 contain those chromosomes whose remodeling have shaped the karyotypes in flatfish from n = 24 in *P. olivaceus* to 22 *S. maximus* and 21 in *S. senegalensis and C. semilaevis*. A fusion model envisaged suggests a small number of chromosomes in the older lineage Paralichthyidae (9,14 and 16)^10^ that combined with other chromosomes in a lineage-specific way could explain the major rearrangement events that shaped the karyotype in this species.

In conclusion, this study reports a new genome assembly for a male sole and a high-density SNP genetic map with 15,511 high-quality markers distributed in 21 linkage groups. The physical map was anchored to the consensus genetic map to generate 21 pseudo-chromosomes, in agreement with the number of chromosomes in this species. The larger map in females was the result of higher RR with distinct recombination landscape between sexes. Recombination frequencies were used to assess the putative morphology of SseLGs that will have to be validated by cytogenetic studies. A GWAS analysis identified 30 sex-associated markers, all located in SseLG18. A low recombining hot region hosted the putative candidate gene *fshr*. In silico comparison with other Pleuronectiformes genomes demonstrated a high conservation of chromosome synteny, although with much intrachromosomal reorganization. Moreover, these changes in karyotype chromosome number were associated with lineage-specific Robertsonian fusions (i.e. SseLG1 in *S. senegalensis*) and several other rearrangements that involved mainly three chromosomes in the ancestral lineage. The consistent physical and genetic maps reported in Senegalese sole represent a valuable genomic resource for functional and genome-wide association studies, and the identification of genomic processes involved in speciation.

## Supplementary Information

Supplementary Legends.

Supplementary Figure 1.

Supplementary Figure 2.

Supplementary Figure 3.

Supplementary Figure 4.

Supplementary Figure 5.

Supplementary Figure 6.

Supplementary Figure 7.

Supplementary Table 1.

Supplementary Table 2.

Supplementary Table 3.

Supplementary Table 4.

Supplementary Table 5.

Supplementary Table 6.

Supplementary Information.
