# Supplementary material for: Chromosome anchoring in Senegalese sole (Solea senegalensis) reveals sex-associated markers and genome rearrangements in flatfish
Source: Sci Rep. 2021 Jun 29;11:13460. doi: 10.1038/s41598-021-92601-5 (PMC8242048; doi:10.1038/s41598-021-92601-5)
Supplement: Supplementary file 1 — Supplementary Legends. [file 41598_2021_92601_MOESM1_ESM.docx]

**Chromosome anchoring in Senegalese sole (*Solea senegalensis*) reveals sex-associated markers and genome rearrangements in flatfish**

Israel Guerrero-Cózar^1^; Jessica Gomez-Garrido^2^; Concha Berbel^1;^ Juan F. Martinez-Blanch^3^; Tyler Alioto^2,4^; M. Gonzalo Claros^5,6,7,8^; Pierre-Alexandre Gagnaire^9^; Manuel Manchado^1,10*^

^1^IFAPA Centro El Toruño, Junta de Andalucía, Camino Tiro Pichón s/n, 11500 El Puerto de Santa María, Cádiz, Spain

^2^CNAG-CRG, Centre for Genomic Regulation (CRG), Barcelona Institute of Science and Technology (BIST), Barcelona, 08028, Spain.

^3^LifeSequencing, Parc Cientific Universidad De Valencia, Edif. 2, C/ Catedrático Agustín Escardino Benlloch, 9, 46980 Paterna, Spain

^4^Universitat Pompeu Fabra (UPF). 08003 Barcelona, Spain

^5^Universidad de Málaga, Department of Molecular Biology and Biochemistry, Málaga, E-29071, Spain

^6^CIBER de Enfermedades Raras (CIBERER), Málaga, E-29071, Spain

^7^Institute of Biomedical Research in Málaga (IBIMA), IBIMA-RARE, Málaga, E-29010, Spain

^8^Instituto de Hortofruticultura Subtropical y Mediterránea (IHSM-UMA-CSIC), Málaga, E-29010, Spain.

^9^ISEM, Univ Montpellier, CNRS, EPHE, IRD, Montpellier, France

^10^ “Crecimiento Azul", Centro IFAPA El Toruño, Unidad Asociada al CSIC”

*Corresponding author:

Manuel Manchado. IFAPA Centro *El Toruño*. Camino Tiro de Pichón s/n. 11500 El Puerto de Santa María (Cádiz), Spain. Tel: +34 671532088. Fax: +34 856102033. Email: [manuel.manchado@juntadeandalucia.es](mailto:carlos.infante@juntadeandalucia.es)

**Supplementary Fig. S1. Data filtering of markers with Mendelian errors** (available as a separate pdf file).

(A) Data by family (F). The % markers with Mendelian errors in each family are indicated on the bars. (B) Relationship between number of markers with Mendelian errors and % individuals identified. Markers with an error % higher than the threshold (10% in dashed red line) were removed; (C) Relationship between the number of individuals and number of markers with Mendelian errors. Individuals with more than 5% of markers with Mendelian were removed (dashed red line).

**Supplementary Fig. S2. Relationship between physical and genetic distances for linkage groups (SseLG) in male**. (available as a separate pdf file).

The square below indicates the specific recombination landscape.

**Supplementary Fig. S3. Relationship between physical and genetic distances for linkage groups (SseLG) in female** (available as a separate pdf file).

The square below indicates the specific recombination landscape.

**Supplementary Fig. S4. Plots illustrating the recombination frequency estimates (RFm) for intervals between markers for SseLG using the sex-average genetic map**. (available as a separate pdf file).

RFm values were calculated from both chromosomal extremities (right: red circles; left: blue circles), using each of the two terminal markers as a reference starting point.

**Supplementary Fig. S5. Sex-associated SNPs and gene distribution in SseLG18.** (available as a separate excel file).

Significant SNPs using seven families are indicated in red and those significant by family are indicated in green. The gene names and distribution through the SseLG18 are shown. The hot region and the gene candidate *fshr* are indicated

**Supplementary Fig. S6. Dot plot alignment of *S. maximus vs C. semilaevis* (panel left), *S. maximus* *vs P. olivaceus* (panel center) and *P. olivaceus vs C. semilaevis* (panel right).** (available as a separate pdf file).

Chromosome numbers are indicated. The chromosome fusions are boxed. Identity scale is indicated below.

**Supplementary Fig. S7. Synteny between SseLG1, SseLG2 and SseLG3 and the genomes of the flatfish *C. semilaevis* (top), *S. maximus* (center) and *P. olivaceus* (bottom).** (available as a separate pdf file).

Pictures were obtained after syntenic block identification using Satsuma and visualization using Mizbee

**Supplementary Method. Methodology for genome annotation** (available as a separate word file).

**Supplementary Table S1. Main features of ONT and Illumina libraries used in this study.** (available as a separate word file).

Library names, status and the number of reads, average length, total megabases (Mb) and average quality of raw data and after ONT trimming are indicated

**Supplementary Table S2. Summary statistics for ddRAD** (available as a separate excel file).

Sample name, family, number of sequences, average length, total megabases and average quality are indicated.

**Supplementary Table S3. Genome Gene Ontology (GO) annotation.** available as a separate excel file).

Orthology between male and female genes (tab "Female_male_orthology", GO annotation (tab "Full_GOannotation") and sex-specific GO categories (tabs "Female unique" and "Male unique")

**Supplementary Table S4. Summary statistics for male assembly using MaSuRCa and after seven rounds of Pilon.** (available as a separate word file).

For comparison purposes, the main features for a female sole genome recently published are also shown.

**Supplementary Table S5. Significant sex-associated markers after GWAS analysis using 7 families (whole Population) or in separate families (F1, F3, F4, F5 and F8).** (available as a separate excel file).

The marker name, pseudo-chromosome (Chr) location, major (A1) and minor (A2), nimber of individuals analyzed (N), physical position in the pseudo-chromosome (Mb) and e-values for each marker are indicated. When the same marker was signficant in two families in the family-specific GWAS analysis, the number of individulas in each family separated by "/" is shown. The percentages (%) of heterozygous markers in males (M) and females (F) are indicated.

**Supplementary Table S6.** **Macrosynteny of chromosomes between four flatfish species: *S senegalensis*, *C. semilaevis* (Cse), *S. maximus* (Sma) and *P. olivaceus* (Pol).** (available as a separate word file).

Three comparisons were carried out using as reference the species indicated with asterisk. The chromosome number that matches between species is shown. The shaded SseLG denotes that matching with only one chromosome across the three flatfishes. The number of chromosomes (*n*) in each species is also indicated.
