# Supplementary figures and images for: Chromosome anchoring in Senegalese sole (Solea senegalensis) reveals sex-associated markers and genome rearrangements in flatfish
Source: Sci Rep. 2021 Jun 29;11:13460. doi: 10.1038/s41598-021-92601-5 (PMC8242048; doi:10.1038/s41598-021-92601-5)

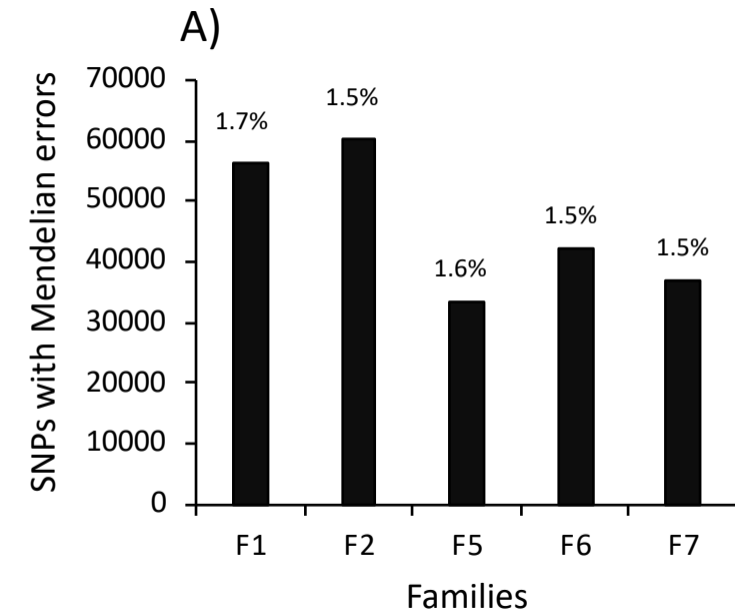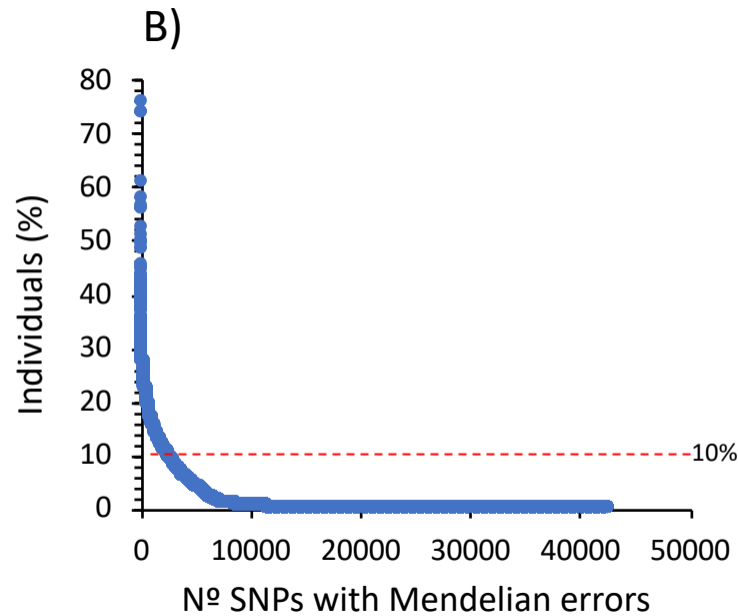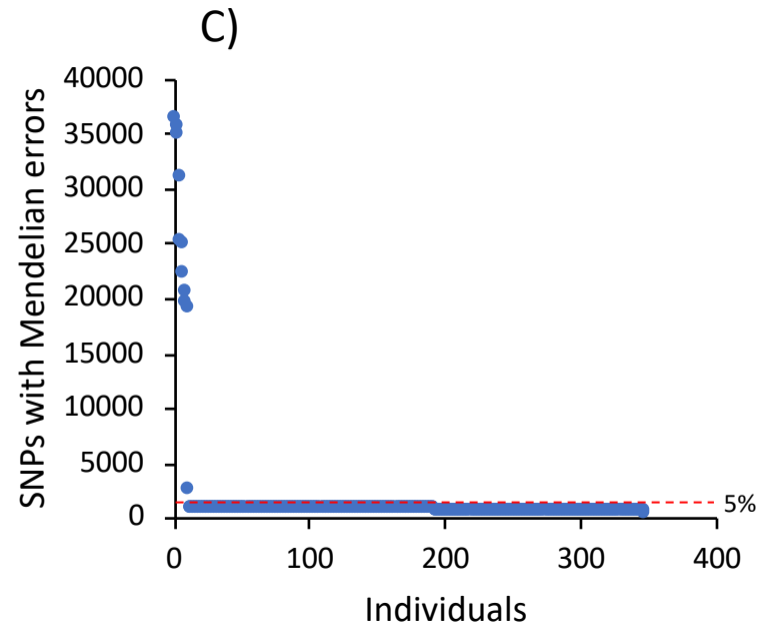

Supplement: Supplementary file 2 — Supplementary Figure 1. [file 41598_2021_92601_MOESM2_ESM.pdf]

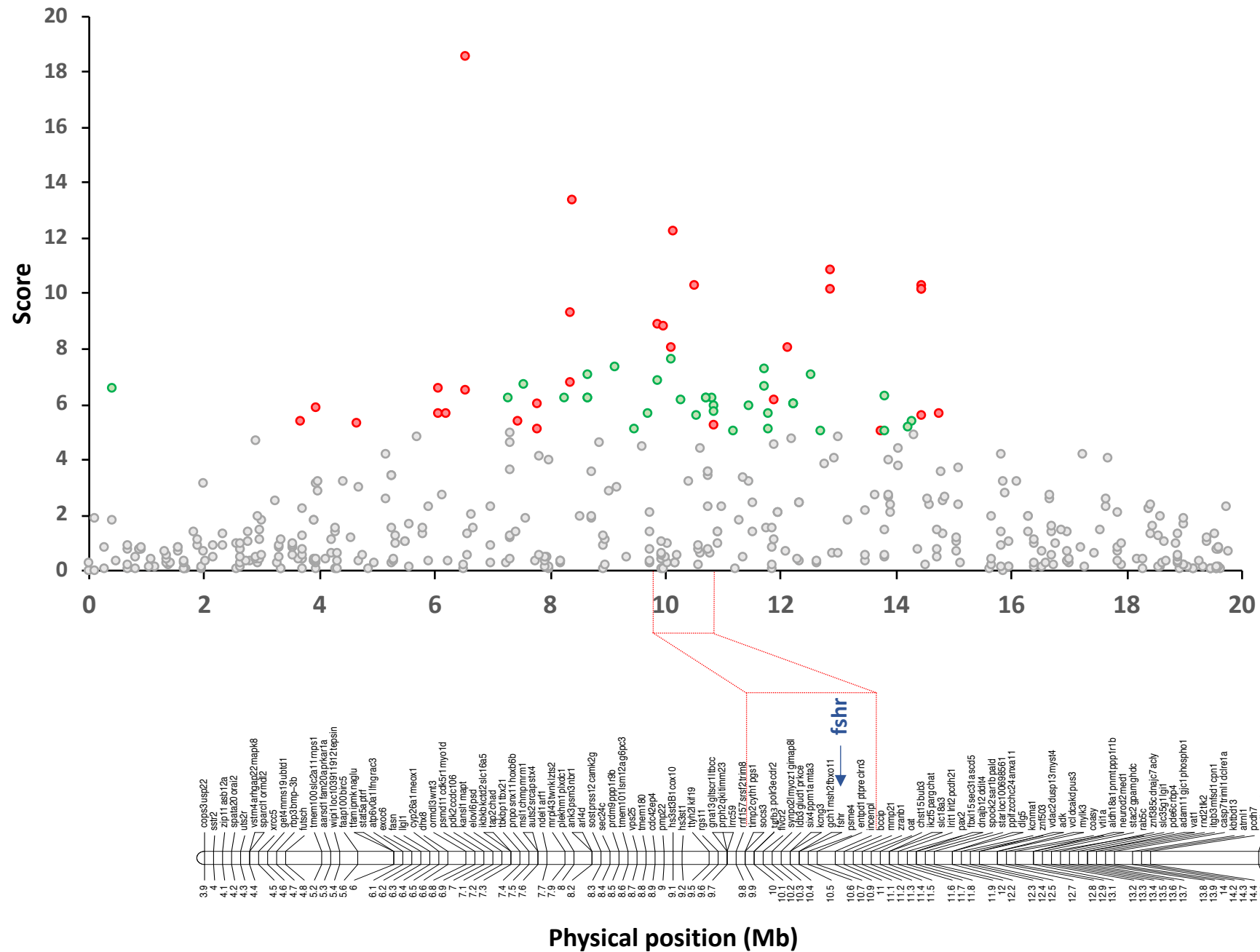

Supplement: Supplementary file 6 — Supplementary Figure 5. [file 41598_2021_92601_MOESM6_ESM.pdf]

*Scophthalmus maximus*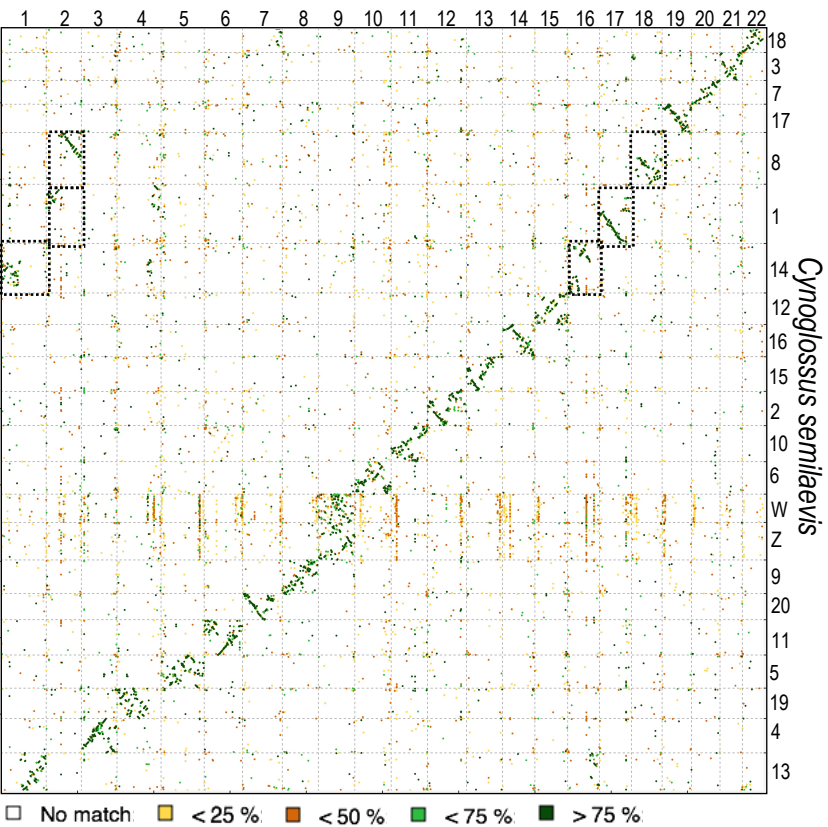*Scophthalmus maximus*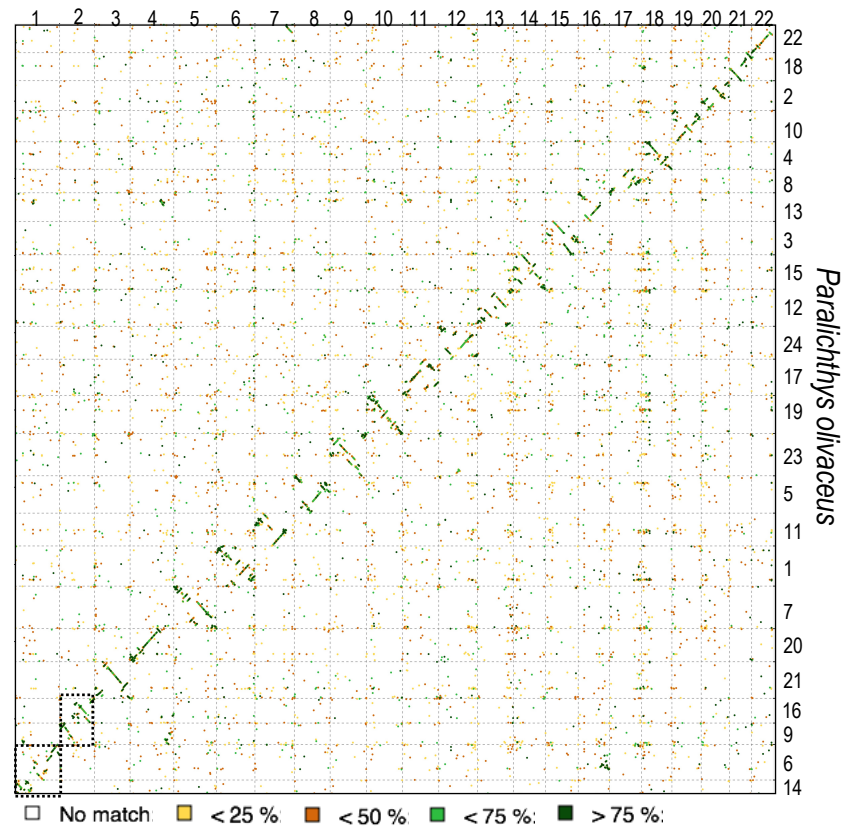*Paralichthys olivaceus*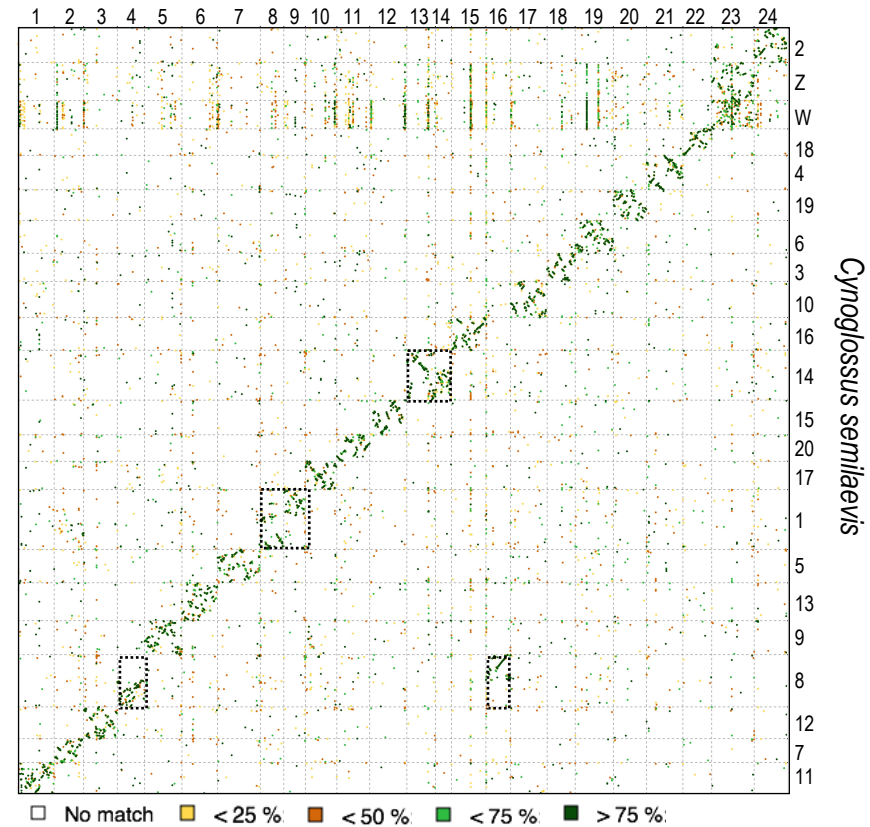

A)

B)

Supplement: Supplementary file 7 — Supplementary Figure 6. [file 41598_2021_92601_MOESM7_ESM.pdf]
