# Supplementary material for: Chromosome anchoring in Senegalese sole (Solea senegalensis) reveals sex-associated markers and genome rearrangements in flatfish
Source: Sci Rep. 2021 Jun 29;11:13460. doi: 10.1038/s41598-021-92601-5 (PMC8242048; doi:10.1038/s41598-021-92601-5)

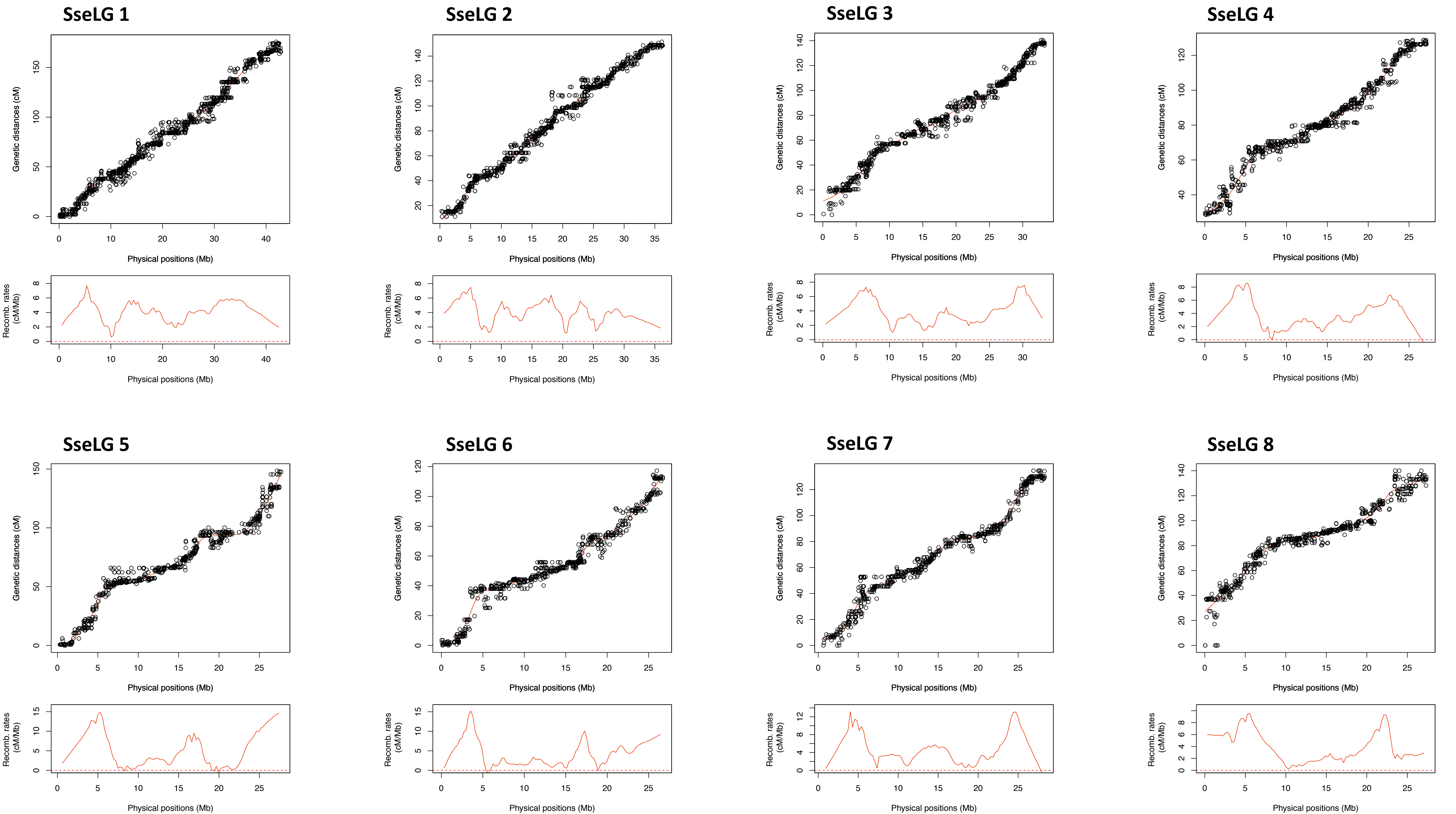
SseLG 1
SseLG 2
SseLG 3
SseLG 4
SseLG 5
SseLG 6
SseLG 7
SseLG 8
Genetic distances (cM)
Physical positions (Mb)
Recomb. rates (cM/Mb)

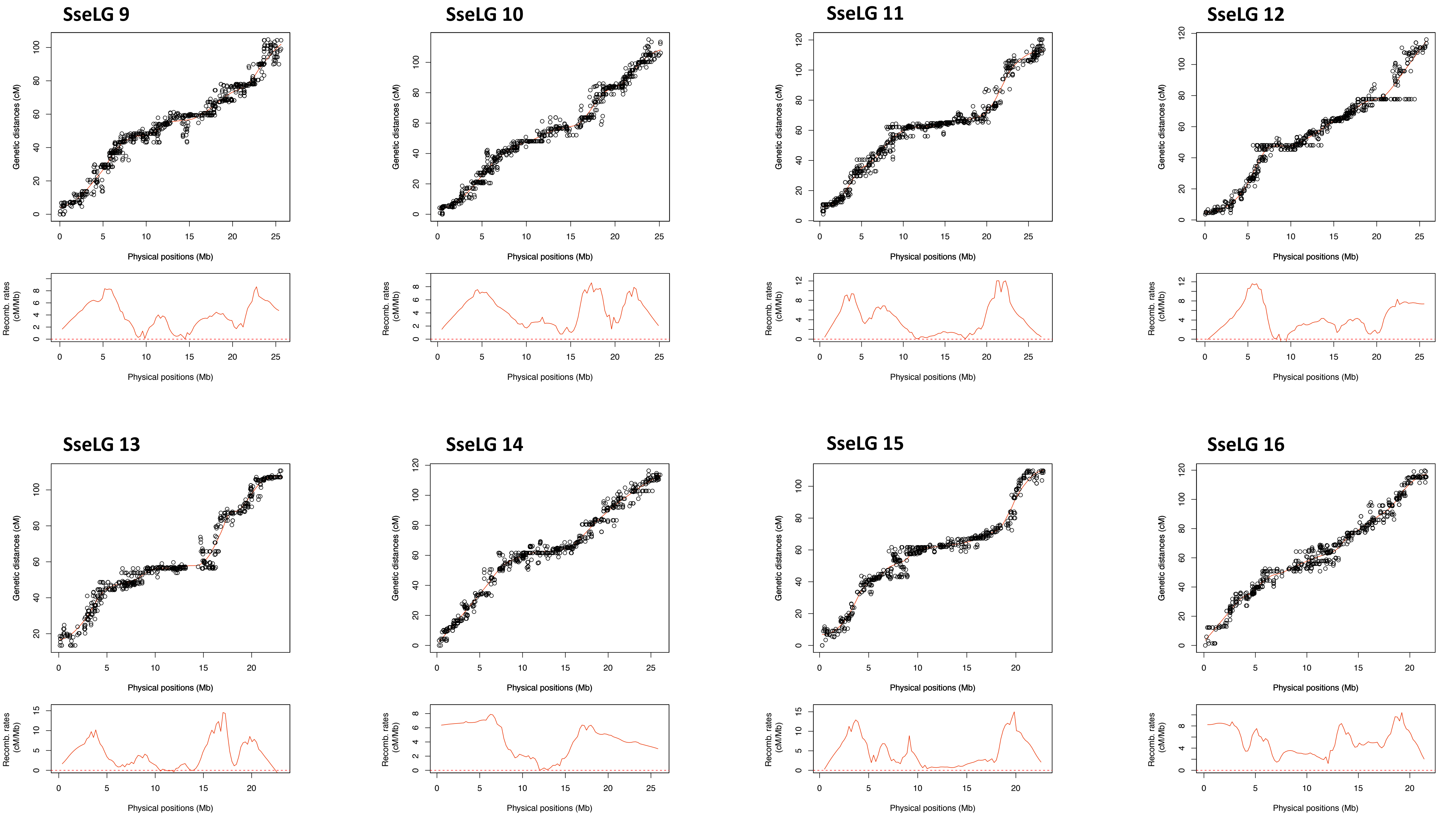
SseLG 9
SseLG 10
SseLG 11
SseLG 12
SseLG 13
SseLG 14
SseLG 15
SseLG 16
Genetic distances (cM)
Physical positions (Mb)
Recomb. rates (cM/Mb)

SseLG 17

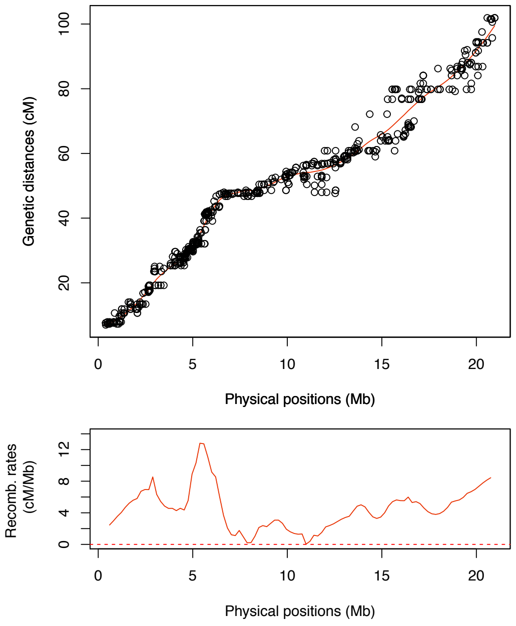

SseLG 18

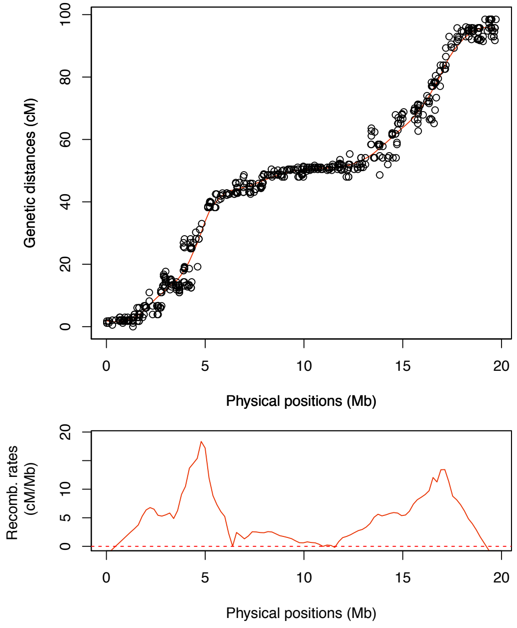

SseLG 19

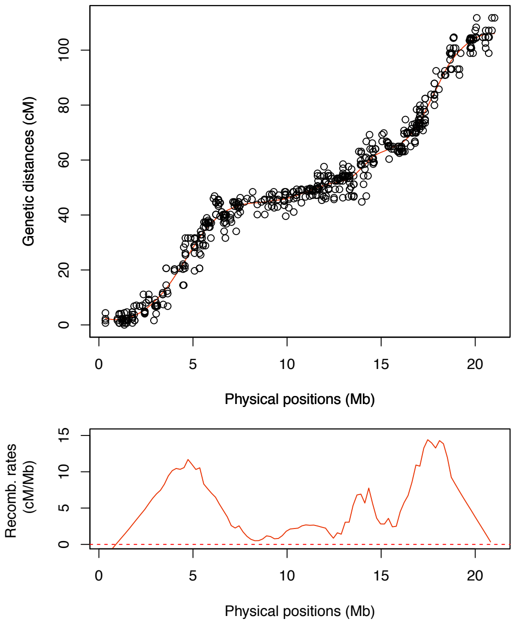

SseLG 20

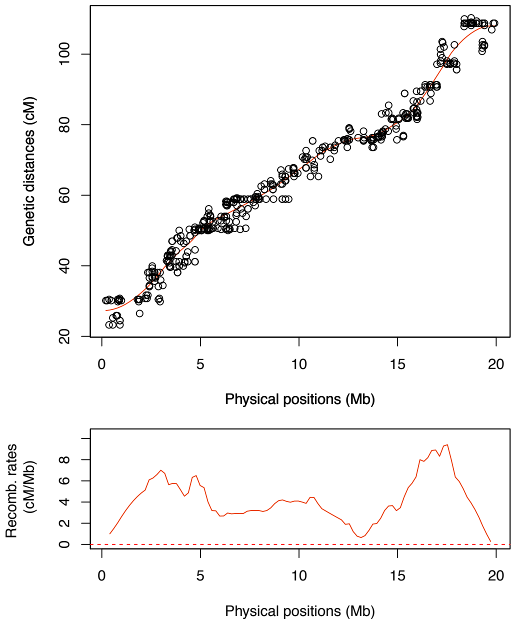

SseLG 21

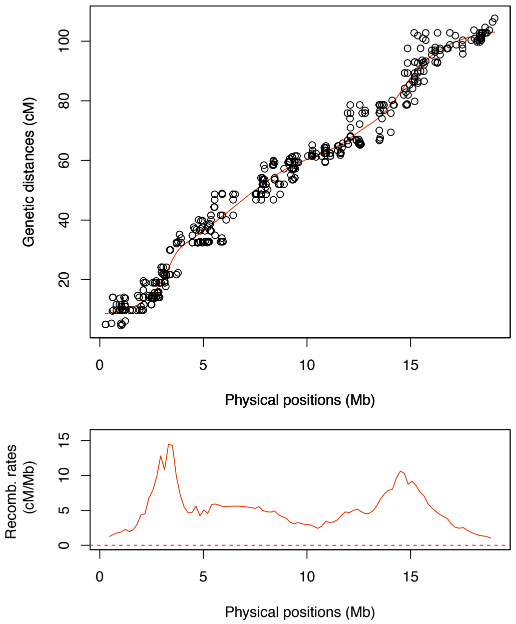

Supplement: Supplementary file 4 — Supplementary Figure 3. [file 41598_2021_92601_MOESM4_ESM.pdf]
