# Supplementary material for: Chromosome anchoring in Senegalese sole (Solea senegalensis) reveals sex-associated markers and genome rearrangements in flatfish
Source: Sci Rep. 2021 Jun 29;11:13460. doi: 10.1038/s41598-021-92601-5 (PMC8242048; doi:10.1038/s41598-021-92601-5)
Supplement: Supplementary file 5 — Supplementary Figure 4. [file 41598_2021_92601_MOESM5_ESM.pdf]

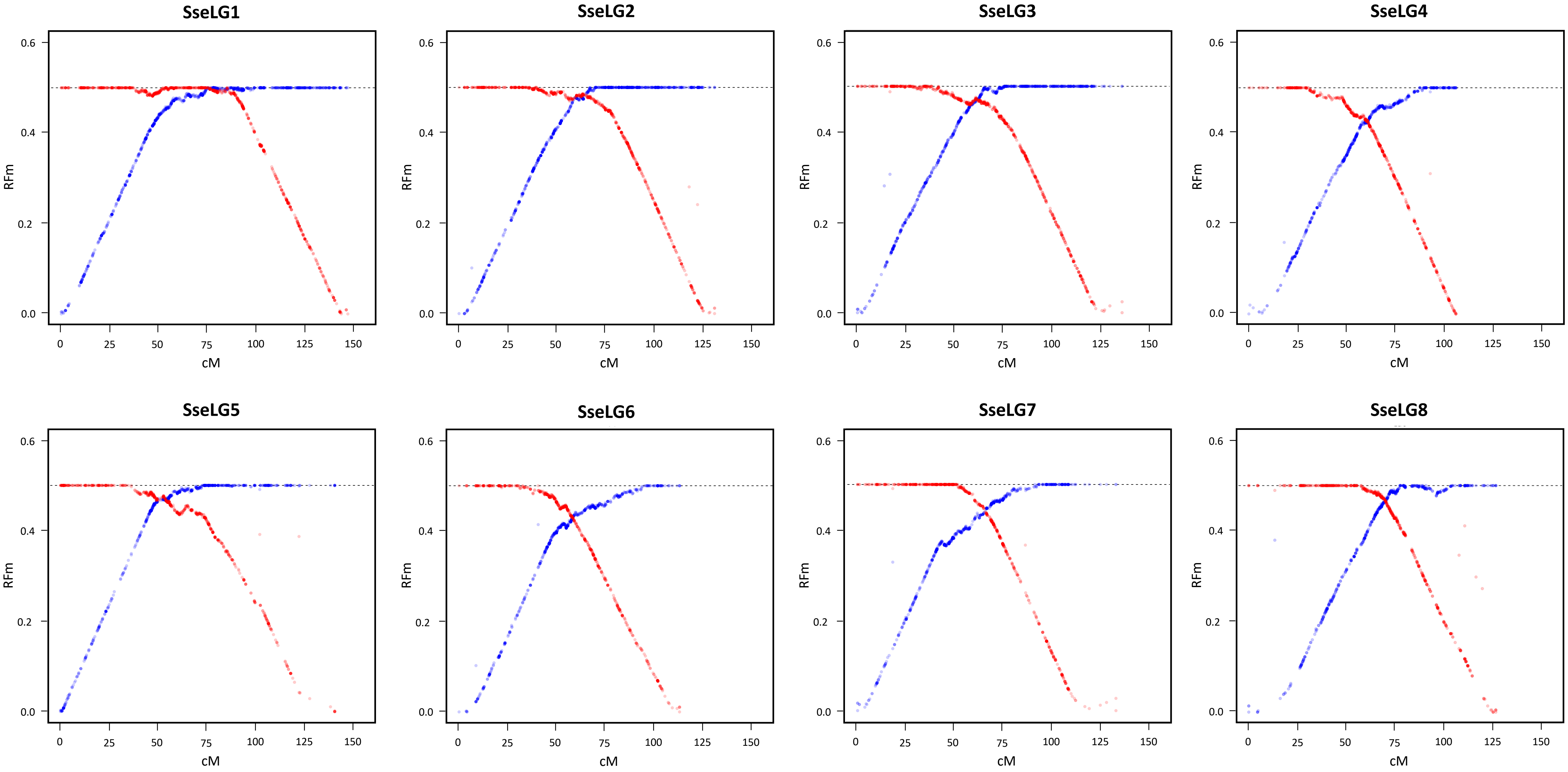
SseLG1
SseLG2
SseLG3
SseLG4
SseLG5
SseLG6
SseLG7
SseLG8
RFm
cM

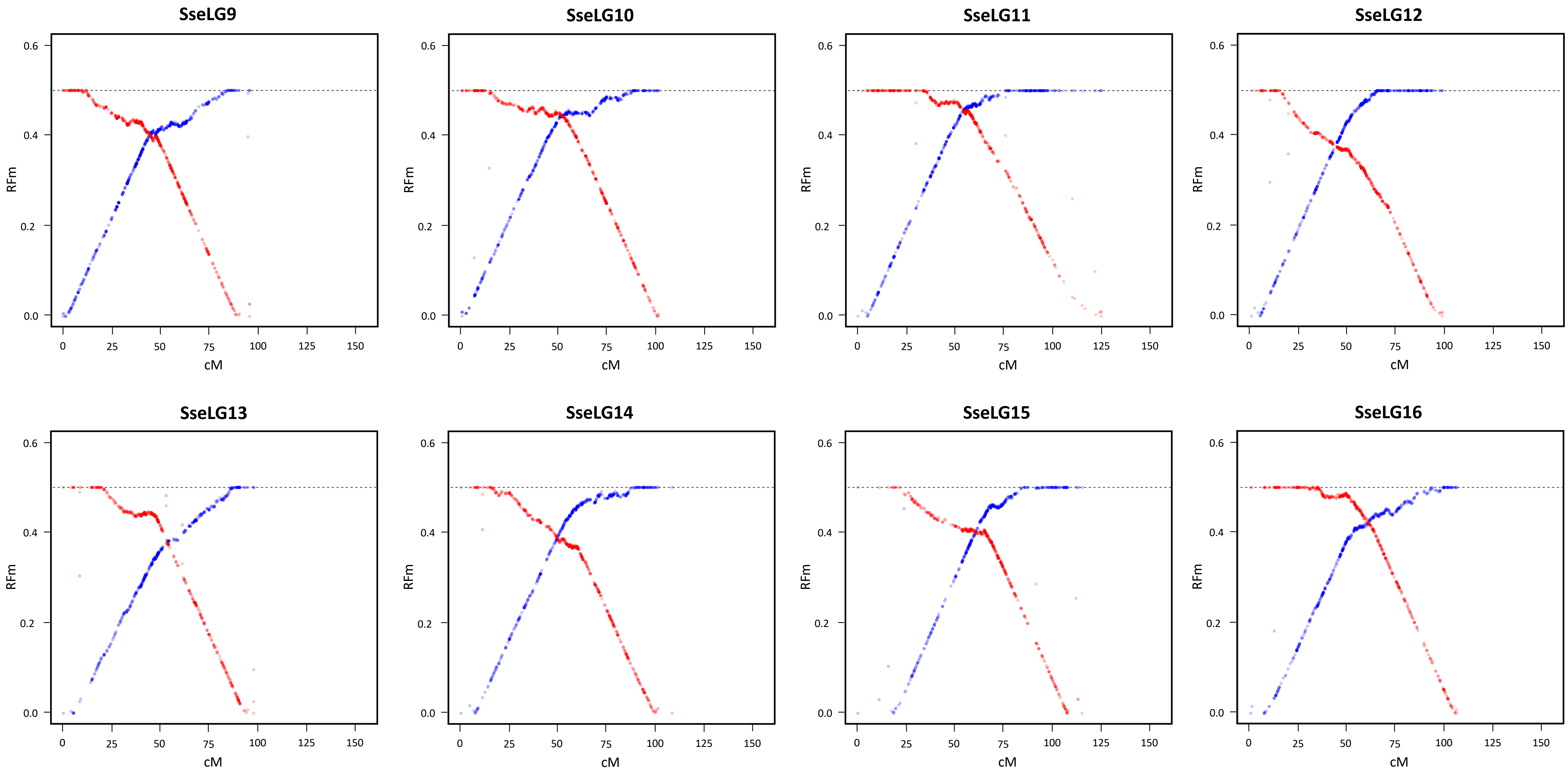
SseLG9
SseLG10
SseLG11
SseLG12
SseLG13
SseLG14
SseLG15
SseLG16
RFm
cM
0.0
0.2
0.4
0.6
0
25
50
75
100
125
150

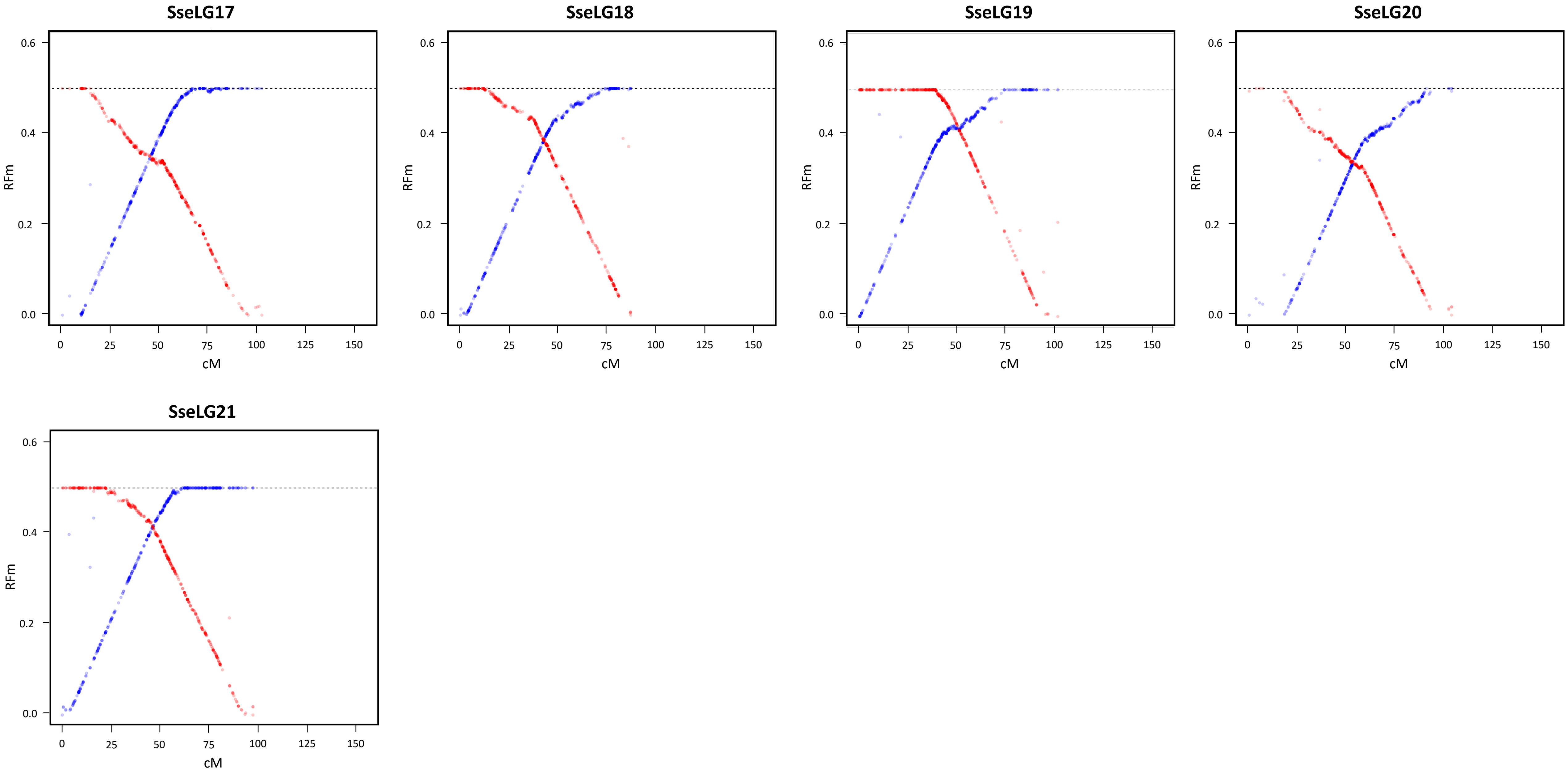
SseLG17
SseLG18
SseLG19
SseLG20
SseLG21
RFm
cM
0.0
0.2
0.4
0.6
0
25
50
75
100
125
150
