# Supplementary material for: Chromosome anchoring in Senegalese sole (Solea senegalensis) reveals sex-associated markers and genome rearrangements in flatfish
Source: Sci Rep. 2021 Jun 29;11:13460. doi: 10.1038/s41598-021-92601-5 (PMC8242048; doi:10.1038/s41598-021-92601-5)
Supplement: Supplementary file 8 — Supplementary Figure 7. [file 41598_2021_92601_MOESM8_ESM.pdf]

A) Whole genome synteny

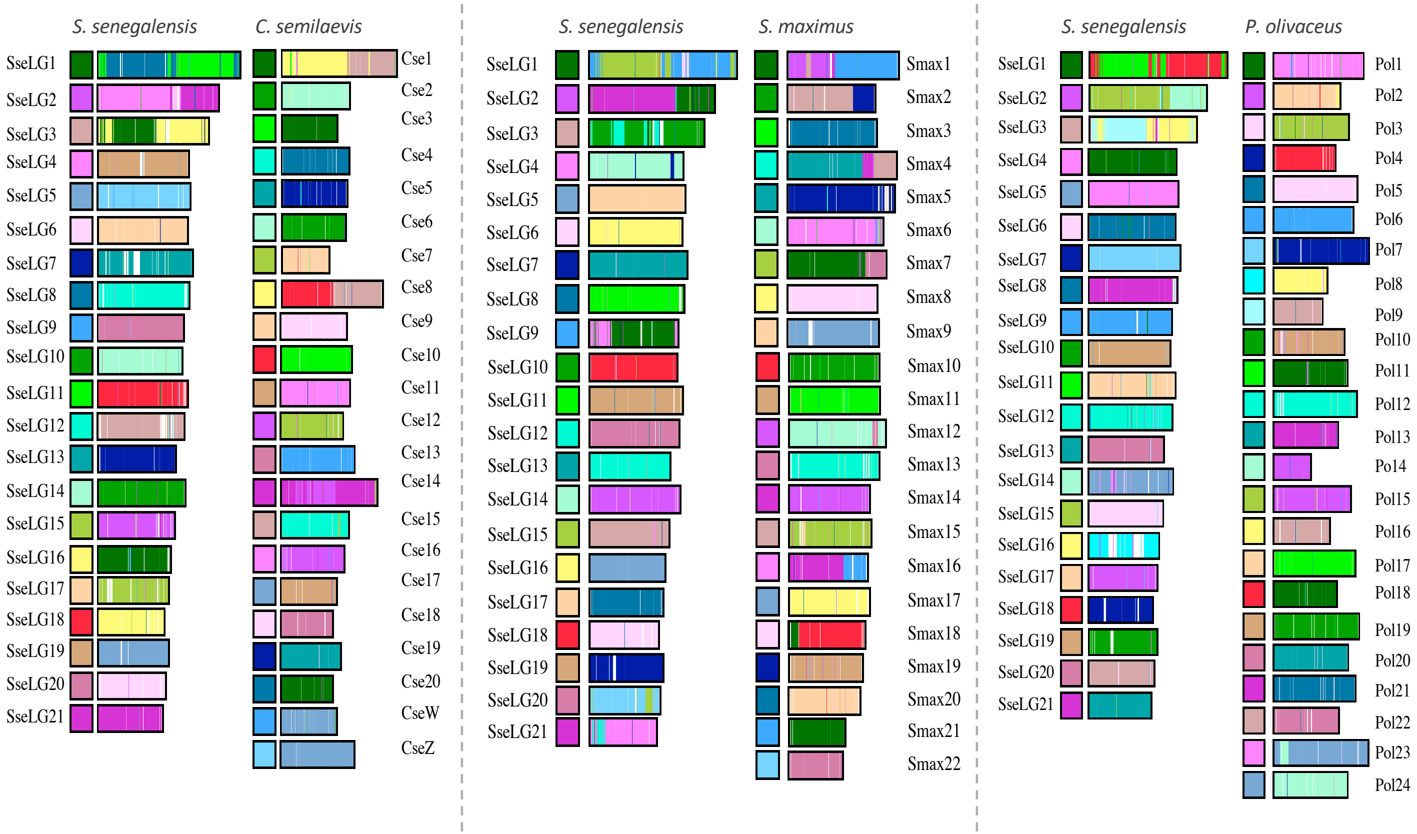

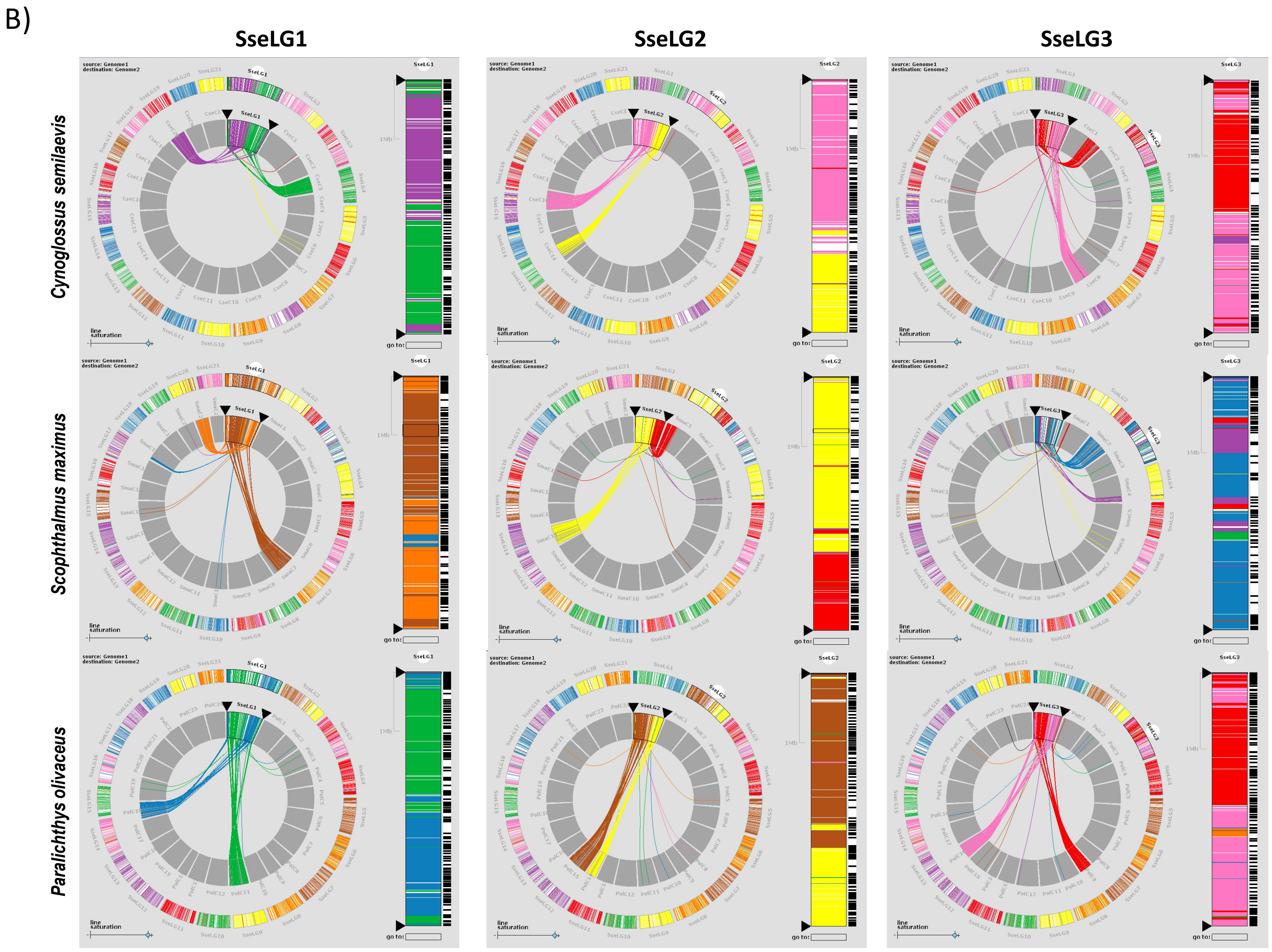
B)
SseLG1
SseLG2
SseLG3
Cynoglossus semilaevis
Scophthalmus maximus
Paralichthys olivaceus
