# Supplementary material for: Chromosome anchoring in Senegalese sole (Solea senegalensis) reveals sex-associated markers and genome rearrangements in flatfish
Source: Sci Rep. 2021 Jun 29;11:13460. doi: 10.1038/s41598-021-92601-5 (PMC8242048; doi:10.1038/s41598-021-92601-5)
Supplement: Supplementary file 9 — Supplementary Table 1. [file 41598_2021_92601_MOESM9_ESM.docx]

**Supplementary Table S1. Main features of ONT and Illumina libraries used in this study.** Library names, status and the number of reads, average length, total megabases (Mb) and average quality of raw data and after ONT trimming are indicated

| ONT Library names | Status | Reads | Av. length | Total Mb | Av. Qual |
| --- | --- | --- | --- | --- | --- |
| P_IFAPA_190280A_raw | pass | 1,082,622 | 4,304,38 | 4,660.02 | 10.77 |
| P_IFAPA_190280A_raw | fail | 633,506 | 3,313.31 | 2,099.00 | 6.98 |
| P_IFAPA_187531A_raw | pass | 1,236,171 | 4,693.36 | 5,801.80 | 11.16 |
| P_IFAPA_187531A_raw | fail | 541,313 | 3,770.66 | 2,041.11 | 6.87 |
| P_IFAPA_187531-1_raw | pass | 1,088,666 | 4,260.95 | 4,638.76 | 11.24 |
| P_IFAPA_187531-1_raw | fail | 487,091 | 3,282.75 | 1,599.00 | 6.88 |
| P_IFAPA_187531-2_raw | pass | 945,387 | 4,343.72 | 4,106.49 | 10.94 |
| P_IFAPA_187531-2_raw | fail | 498,841 | 3,417.80 | 1,704.94 | 6.93 |
| ONT libraries after trimming | | | | | |
| P_IFAPA_190280A_trimmed |  | 1,091,637 | 4,241.64 | 4,630.33 | 10.80 |
| P_IFAPA_187531A_trimmed |  | 1,249,034 | 4,641.08 | 5,796.86 | 11.19 |
| P_IFAPA_187531-1_trimmed |  | 1,099,312 | 4,217.90 | 4,636.78 | 11.27 |
| P_IFAPA_187531-2_trimmed |  | 952,030 | 4,300.36 | 4,094.07 | 10.97 |
| Illumina Library names |  |  |  |  |  |
| run2_0510M R1 |  | 143,602,543 | 148.08 | 21,264.81 | 30.77 |
| run2_0510M R2 |  | 143,602,543 | 148.09 | 21,265.63 | 30.12 |
