# Supplementary material for: Chromosome anchoring in Senegalese sole (Solea senegalensis) reveals sex-associated markers and genome rearrangements in flatfish
Source: Sci Rep. 2021 Jun 29;11:13460. doi: 10.1038/s41598-021-92601-5 (PMC8242048; doi:10.1038/s41598-021-92601-5)
Supplement: Supplementary file 12 — Supplementary Table 4. [file 41598_2021_92601_MOESM12_ESM.docx]

**Supplementary Table S4. Summary statistics for male assembly using MaSuRCa and after seven rounds of Pilon.** For comparison purposes, the main features for a female sole genome recently published are also shown.

|  | Male assembly | | Female^#^ |
| --- | --- | --- | --- |
|  | MaSuRCa | Pilon |  |
| Contigs (>= 0 bp) | 3,403 | 3,403 | 5,748 |
| Contigs (>=10 kb) | 2,768 | 2,754 | 3,890 |
| Contigs (>=25 kb) | 2,064 | 2,060 | 2,837 |
| Contigs (>=50 kb) | 1,686 | 1,682 | 2,233 |
| Total length (>= 0 bp) | 610,444,213 | 609,359,514 | 607,976,531 |
| Total length (>= 10 kb) | 606,748,022 | 605,636,885 | 599,101,686 |
| Total length (>= 25 kb) | 595,278,851 | 594,291,065 | 581,919,585 |
| Total length (>= 50 kb) | 581,780,278 | 580,751,380 | 560,354,333 |
| Largest contig | 4,530,339 | 4,527,104 | 1,681,779 |
| Total length | 610,444,213 | 609,359,514 | 607,976,531 |
| GC (%) | 40.92 | 40.92 | 40.90 |
| N50 | 508,691 | 512,746 | 339,942 |
| N75 | 245,352 | 245,130 | 167,779 |
| L50 | 313 | 313 | 548 |
| L75 | 743 | 743 | 1177 |
| N’s per 100 kbp | 1.34 | 1.21 | 157.3 |

^#^ Sequence deposited in figshare https://doi.org/10.6084/m9.figshare.12472100.v1..
