# Supplementary material for: Chromosome anchoring in Senegalese sole (Solea senegalensis) reveals sex-associated markers and genome rearrangements in flatfish
Source: Sci Rep. 2021 Jun 29;11:13460. doi: 10.1038/s41598-021-92601-5 (PMC8242048; doi:10.1038/s41598-021-92601-5)
Supplement: Supplementary file 14 — Supplementary Table 6. [file 41598_2021_92601_MOESM14_ESM.docx]

**Supplementary Table S6**. Chromosome correspondence according to synteny and dot-plot alignment: *S senegalensis*, *C. semilaevis* (Cse), *S. maximus* (Sma) and *P. olivaceus* (Pol) between four flatfish species. Three comparisons were carried out using as reference the species indicated with asterisk. The chromosome number that matches between species is shown. Shading denotes a one-to-one matching between SseLGs and chromosomes across the three flatfishes. The number of chromosomes (*n*) in each species is also indicated.

| *S. senegalensis (n=21)** | *Cse^#^ (n=21)* | *Sma^‡^ (n=22)* | *Pol^†^ (n=24)* |  | *Cse** | *Sma* | *Pol* |  | *Sma** | *Pol* |
| --- | --- | --- | --- | --- | --- | --- | --- | --- | --- | --- |
| SseLG1 | 3,20 | 7,21 | 11,18 |  | Chr1 | 17,2 | 8,9 |  | Chr1 | 14,6 |
| SseLG2 | 14,16 | 1,14 | 14,15 |  | Chr2 | 12 | 24 |  | Chr2 | 9,16 |
| SseLG3 | 8,1 | 2,4 | 9,16 |  | Chr3 | 21 | 18 |  | Chr3 | 21 |
| SseLG4 | 11 | 6 | 1 |  | Chr4 | 3 | 21 |  | Chr4 | 20 |
| SseLG5 | Z/W | 9 | 23 |  | Chr5 | 5 | 7 |  | Chr5 | 7 |
| SseLG6 | 9 | 13 | 5 |  | Chr6 | 10 | 19 |  | Chr6 | 1 |
| SseLG7 | 5 | 5 | 7 |  | Chr7 | 20 | 2 |  | Chr7 | 11 |
| SseLG8 | 4 | 3 | 21 |  | Chr8 | 2,18 | 16,4 |  | Chr8 | 5 |
| SseLG9 | 13 | 16,1 | 6 |  | Chr9 | 8 | 5 |  | Chr9 | 23 |
| SseLG10 | 6 | 10 | 19 |  | Chr10 | 11 | 17 |  | Chr10 | 19 |
| SseLG11 | 10 | 11 | 17 |  | Chr11 | 6 | 1 |  | Chr11 | 17 |
| SseLG12 | 15 | 13 | 12 |  | Chr12 | 15 | 3 |  | Chr12 | 24 |
| SseLG13 | 19 | 4 | 20 |  | Chr13 | 1 | 6 |  | Chr13 | 12 |
| SseLG14 | 2 | 12 | 24 |  | Chr14 | 16,1 | 13,14 |  | Chr14 | 15 |
| SseLG15 | 12 | 15 | 3 |  | Chr15 | 13 | 12 |  | Chr15 | 3 |
| SseLG16 | 1 | 17 | 8 |  | Chr16 | 14 | 15 |  | Chr16 | 13 |
| SseLG17 | 7 | 20 | 2 |  | Chr17 | 19 | 10 |  | Chr17 | 8 |
| SseLG18 | 8 | 18 | 4 |  | Chr18 | 22 | 22 |  | Chr18 | 4 |
| SseLG19 | 17 | 14 | 10 |  | Chr19 | 4 | 20 |  | Chr19 | 10 |
| SseLG20 | 18 | 7,22 | 22 |  | Chr20 | 7 | 11 |  | Chr20 | 2 |
| SseLG21 | 14 | 16,4 | 13 |  | ZW | 9 | 23 |  | Chr21 | 18 |
|  |  |  |  |  |  |  |  |  | Chr22 | 22 |

*^#^Assembly Acc. No GCF_000523025.1; ^‡^ Assembly Acc. No: GCA_013347765.1; ^†^Assembly Acc. No: GCA_001904815.1*
