# Supplementary material for: Chromosome anchoring in Senegalese sole (Solea senegalensis) reveals sex-associated markers and genome rearrangements in flatfish
Source: Sci Rep. 2021 Jun 29;11:13460. doi: 10.1038/s41598-021-92601-5 (PMC8242048; doi:10.1038/s41598-021-92601-5)
Supplement: Supplementary file 15 — Supplementary Information. [file 41598_2021_92601_MOESM15_ESM.docx]

## Full Methods

## 1. Genome sequencing and assembly

High-molecular weight genomic DNA was prepared from heparinized whole blood using the MagAttract HMW DNA kit (Qiagen). Sample was quantified using the Nanodrop ND-8000 and checked for quality on a 0.8% agarose gel before sending to the company LifeSequencing S.L. (Valencia, Spain) for library preparations. This company confirmed DNA quantification with the Qubit DNA assay (ThermoFisher Sci.) and checked integrity using the Genomic DNA ScreenTape kit on an Agilent 4200 TapeStation. Selected sampled for sequencing had OD_260_/OD_280_ and OD_260_/OD_230_ ratios higher than 1.7, DIN >8.0 and average DNA size >50 kb. Long-read sequencing was carried out using the Oxford nanopore Technology (ONT) MinION platform. Four libraries were prepared for sequencing using the protocol 1D^2^ library preparation SQK-LSK309 kit (version LSD_9097_v12), sequenced on R9.5 flow cells for 36 hours each, following the manufacturer's recommendations (Oxford Nanopore). Overall, 19.2 Gb of genome information was generated with an average read length of 4.3 kb. An aliquot of the same high-molecular weight genomic DNA was sent to the genomic service unit of the University of Malaga to be sequenced on an Illumina platform. Two libraries were constructed using standard kit reagents from the Nextera DNA Flex Library Prep kit (Illumina, USA) following the manufacturer’s protocol. Average size of library fragments was 716 bp. Libraries were sequenced on a NextSeq550 sequencer (Illumina, USA) that overall generated 43 Gb of sequence from 143 million reads (average length 147 nt). The main features of the libraries used during the genome assembly are presented in Supplementary Table 1. The raw read data were deposited to the NCBI Sequence Read Archive (SRA) under accession number SAMN16809702.

Before assembling the genome, Illumina reads were checked with FastQC v0.11.5 for quality and sequencing adapters were trimmed using cutadapt v1.4.1. Moreover, long ONT reads were base-called with Albacore v2.0.2 and reads with an average quality lower than Q9 were discarded. Adapters were removed using Porechop*v*0.2.4 ^1^. The hybrid genome assembly was carried out using MaSuRCA*v*3.2.3 ^2,3^ with the Illumina libraries (57.3x coverage) and the error-corrected Nanopore reads (25.5x). The LR-hybrid assembly was characterized for completeness using Benchmarking Universal Single-Copy Orthologs (BUSCO*v*3.0.2)^4,5^ containing 4,854 single-copy orthologs from actinopterygii_odb9. Resulting scaffolds were finally polished using Pilon, v1.23 ^6^ with high-quality Illumina reads (Q20, L50) that were filtered with the BBtools software suite (https://sourceforge.net/projects/bbmap/).

## 2. ddRAD-seq library preparation and sequencing

Genomic DNA from the caudal fin (offspring) or whole blood (parents) were purified using the Isolate II Genomic DNA Kit (Bioline). DNA samples were treated with RNase A (Bioline) following the manufacturer’s protocol. DNA was quantified spectrophotometrically using the Nanodrop ND-8000. The OD_260_/OD_280_ ratio ranged between 1.8 and 2.0 the OD_260_/OD_230_ between 2.0 and 2.2. The integrity of DNA was assessed by agarose gel electrophoresis checking that a major band between 8-10 kb could be observed.

DNA was sent to the company LifeSequencing S.L. (Valencia, Spain) to prepare the ddRAD libraries. DNA quantification was confirmed with the Quant-iT PicoGreen dsDNA Assay Kit (Thermofisher Sci) and read in a fluorometer Qubit 2.0 (Invitrogen^®^). DNA integrity was evaluated using the Genomic DNA ScreenTape Kit on an Agilent 4200 TapeStation. A total of 346 samples passed all quality controls and were subsequently used for library construction (Table 1). To prepare the ddRAD sequencing libraries, a preliminary trial to select the optimal enzyme combination to produce the highest number of SNPs was carried out using 13 wild fish including the eight parents of the five selected families. Two enzyme combinations were tested: SbfI (specific for the CCTGCA|GG motif) and SphI (specific for the GCATG|C motif) and SbfI and NcoI (specific for the GGTAC|C motif) as previously described by Brown et al. ^7^. Libraries were constructed based on the protocol described by Peterson et al. ^8^. Briefly, DNA (200 ng) was digested with restriction enzymes (8 U per reaction) at 37ºC for 4 h followed by 80ºC at 20 min for inactivation. Thereafter, adapters were ligated by incubating at 23ºC for 120 min with T4 DNA ligase (200 U/reaction, NEB) and 200 nM of each primer including Illumina specific sequence for indexing. Reactions were inactivated at 65ºC for 20 min and fragments were purified and concentrated using AMPure XT beads (Beckman Coulter Genomics, USA). Individual libraries were amplified using Dual index Nextera XT Kit (Illumina) following the manufacturer's instructions. Libraries were quantified by fluorimetry (Quant-iT PicoGreen dsDNA Assay Kit, Thermofisher Sci) and equal amounts of purified PCR products (40 nM) were pooled and subjected to size selection (350-700 nt) using a 2% agarose. Final pools were analysed using an Agilent 2100 Bioanalyzer in order to check primer-dimer elimination and sized selection confirmation. The pool of libraries was loaded on a Nextseq 500 sequencer (Illumina), following the manufacturer's instructions for 150 paired-end reads and spiking 20% of PhiX Sequencing Control V3 (Illumina). Bioinformatic analysis was carried as described below. Both enzyme combinations showed overall less than 20,000 SNPs per sample (SbfI-NcoI; 15,597 SNPs and SbfI-SphI; 13,070 SNPs). In order to enhance the total number of SNPs per sample, SbfI was substituted by EcoRI (CTTAA|G) following the recommendations in Peterson *et al.,* (2012) (shorter enzymatic restriction site, 6 *vs* 8 nt and lower GC content, 33 *vs* 66%). This new enzyme combination EcoRI/NcoI generated an average of 24,874 SNPs per sample. Once the conditions were optimized, all DNA samples (Table 1) were processed to construct the ddRAD libraries. Pools of libraries were loaded on a Novaseq 6000 sequencer (Illumina), following the manufacturer's instructions and the specifications mentioned above. The total number of reads generated for each library are indicated in Supplementary Table 2.

## 3. Reads filtering and SNP calling

Illumina reads were processed using Stacks v2.3e^9^, a pipeline specifically designed for RAD data analysis. First, the process_radtags function was used to demultiplex individual samples. During this step sequence reads with quality scores below 10, missing either restriction site or with ambiguous barcodes were discarded and all sequences were trimmed to 147 nt long. Filtered data were then aligned to male genome generated in this study. Stacks *v*2 uses a de Bruijn graph assembler to build and connect contigs from forward and reverse reads for each *de novo* RAD locus, which is then used as a reference for read alignments. The key parameter values used to identify RAD *loci* were clustering threshold of 2 (D), which is the maximum distance between ddRAD sequences allowing identification of homologous *loci*, a minimum stack depth (m) of 3, a maximum of 2 mismatches per locus (M) per individual and up to 1 mismatch between homozygous *loci* from different individuals when building the catalog (n). A final filtering step was carried out using the populations program with the option –r = 0.80, in order to retain only those *loci* represented in at least 80% of the whole population. Rarefaction curves, density plots of locus and SNPs were performed from file catalog.calls in a VCF format with software R version 3.2.3.

## 4 Genetic linkage map

To build the map, SNPs were filtered using Plink v1.9^10^ and those markers that segregated with Mendelian errors in more than 10% of individuals and those individuals with more than 5% of markers with Mendelian errors were removed (Supplementary Fig. 1). The final SNP dataset contained 40,041 markers from 327 individuals (Table 1) and 8 parents that were imported in LepMap3^11^. SNPs were filtered out with ParentCall2 to remove non-informative sites and those markers with segregation distortions using a *P*-value threshold of 0.05. The SNPs were grouped within 21 linkage groups (named as SseLGs) corresponding to the expected number of chromosomes (n=21) using the·"SeparateChromosomes” module. A LOD threshold of 11 and a size limit of 200 were selected as the most adequate parameters to keep an optimal number of markers grouped in the expected number of SseLGs (Fig.1A and B). Module JoinSingles2 was run to assign additional single SNPs to existing SseLG using decreasing LOD score iterations from 10 to 5 (Fig. 1B). Finally, the genetic distances between markers on each SseLG was calculated with the OrderMarkers2 module (male, female, sex average (SA)) using the Kosambi mapping function. The resulting genetic map was visualized using the software linkagemapview^12^.

## 5. Anchoring of genome scaffolds to the genetic linkage map

To obtain a physical map based on 21 pseudo-chromosomes, the male genome assembly generated in this study was anchored to the linkage map using the Lep-Anchor program, following the author's recommendation^13^. Firstly, highly repetitive DNA sequences were masked from the genome assembly using WinMasker (WM) ^14^, and contigs corresponding to homologous haplotypes from a same locus were merged using HaploMerger2 ^15^. Then, the consensus sex-averaged (SA) genetic map and the male genome were processed with the "CleanMap" module that clusters contigs into chromosomes based on mapped markers and cuts chimeric contigs. Thereafter, contigs were sorted and orientated with the "PlaceAndOrientContigs" module to maximize the correlation between physical and linkage maps positions.

**Method for genome annotation**

The genome assemblies were annotated by combining transcript and protein alignments and *ab initio*gene predictions. The annotation process was separately run for the de novo male genome (this study) and the genome previously reported for a female^16,17^, with minor differences that are detailed below. The flowchart of the annotation pipelines is shown in Figure 1 for the male and Fig. 2 for female.

Firstly, repeats were annotated with RepeatMasker *v*4.0.7 (<http://www.repeatmasker.org)> using the custom repeat library available for *S. senegalensis*. In addition, a new repeat library, specific for our male assembly, was built in order to improve the detection of repeats that could interfere in the gene annotation and be absent in the RepBase database. This specific library was the result of running RepeatModeler *v*1.0.11 against the male assembly and excluding those repeats that were part of repetitive protein families (performing a Blast ^18^ against Uniprot) from the resulting library. Finally, the two genomes were re-annotated with RepeatMasker and this repeat-library.

Next, RNA-seq reads from several tissues and developmental stages (Table 1) were downloaded from NCBI and aligned to the assemblies with STAR^19^ (v-2.7.2a). Transcript models were subsequently generated using Stringtie *v*2.0.1)^20^ on each BAM file and then all the models produced were combined using TACO ^21^. High-quality junctions to be used during the annotation process were obtained by running Portcullis^22^ after mapping with STAR. Finally, PASA assemblies were produced with PASA*v*2.3.3^23^ by adding also the transcript sequences of an in-house transcriptome assembly. The *TransDecoder* program, which is part of the PASA package, was run on the PASA assemblies to detect coding regions in the transcripts.

**Table 1:** RNA-seq libraries used for annotation. The tissue or developmental stage, the bioproject when available and the number of samples used are indicated

| Tissue/developmental stage | Bioproject | Samples |
| --- | --- | --- |
| Larvae | PRJNA241068 | 6 |
| Larvae | PRJNA255461 | 4 |
| Larvae | PRJNA261151 | 10 |
| Olfactory rosette | PRJNA319182 | 2 |
| Head-kidney | PRJNA395771 | 9 |
| Eye/brain | PRJNA395771 | 9 |
| Gut/skin | PRJEB29949 | 4 |
| Liver | Internal | 3 |
| Postlarvae | Internal | 2 |

Furthermore, the complete *Danio* *rerio*, *Scophthalmus* *maximus* and *Cynoglosus* *semilaevis* proteomes were downloaded from Uniprot (April 2020) and aligned to the genomes using Spaln v2.4.7^24^. *Ab initio* gene predictions were performed on the repeat masked assemblies with three different programs: GeneID v1.4^25^, Augustus v3.2.3^26^ and Genemark-ES v2.3e^27^ with and without incorporating evidence from the RNA-seq data. The gene predictors were run with trained parameters for human, except Genemark on a self-trained manner. Finally, all the data were combined into consensus CDS models using EvidenceModeler-1.1.1 (EVM)^23^. Additionally, UTRs and alternative splicing forms were annotated through two rounds of PASA annotation updates.

Functional annotation was carried out on the male annotated proteins with Blast2go ^28^. First, a Blastp^18^ search was made against the nr database (last accessed January 2020). Furthermore, Interproscan ^29^ was run to detect protein domains on the annotated proteins. All these data were combined by Blast2go which produced the final functional annotation results.

Before running the protein-coding annotation pipeline in the female assembly, a whole genome alignment of the two assemblies was performed with ProgressiveCactus^30^, using turbot^31^ as an outgroup. The resulting HAL format file was used to run the transMap module of the Comparative Annotation Toolkit (CAT pipeline)^32^ in order to obtain a quick transfer of the genes annotated from the male to the female genome. The resulting GTF file was given as further evidence to EVM during the female annotation process performed through our pipeline (see Fig. 1 and 2). After obtaining the final annotations, a last step to assign gene equivalences between both annotations was carried out. The female proteins inherited then the functional annotation of their male equivalences. Thereafter, the same functional annotation pipeline run in the male genes was also run in the female genes to refine those genes remained as unannotated. Gene Ontology enrichment was performed with topGO in those genes that were unique to one of the genomes.

The annotation of ncRNAs was produced in both assemblies by running the following steps. First, the program cmsearch *v*1.1^33^ that comes with Infernal^34^ was run against the RFAM^35^ database of RNA families (v12.0). Also, tRNAscan-SE v1.23^36^ was run in order to detect the transfer RNA genes present in the genome assembly. The annotated lncRNAs were the result of selecting Pasa-assemblies for those transcripts expressed but not present in the protein-coding gene annotation, that were longer than 200 bp and whose length was not covered at least in an 80% by a small ncRNA. The resulting transcripts were clustered into genes using shared splice sites or significant sequence overlap as criteria for designation as the same gene.

**Figure 1 Male annotation flowchart**


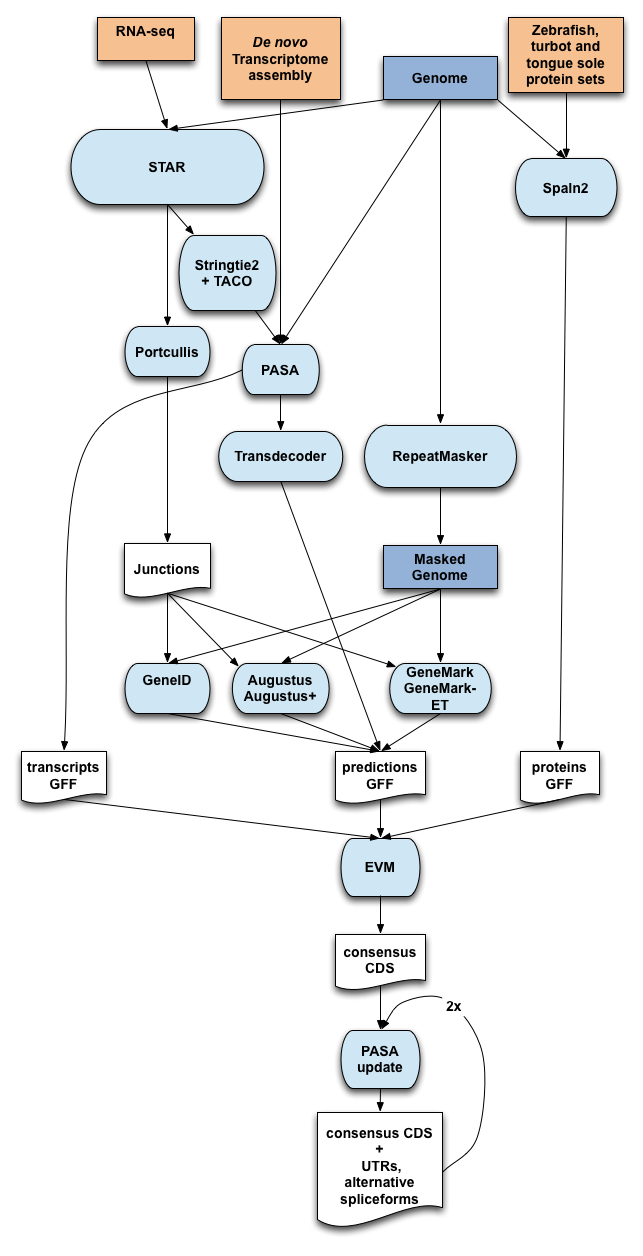


**Figure 2. Female annotation flowchart**


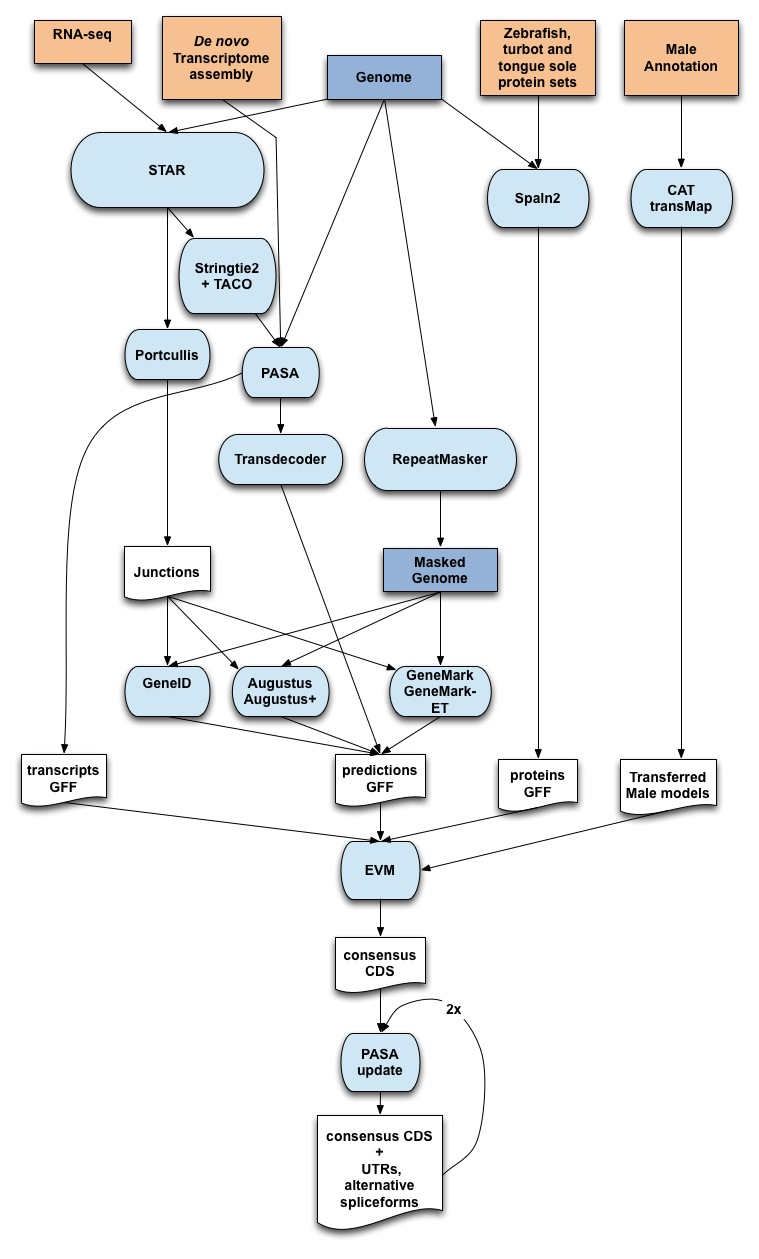


## 6 Recombination rates and cross-species comparisons

Recombination rate variation along the genome was evaluated by comparing the consensus linkage map for both sexes and SA and the physical map of each pseudo-chromosome using MareyMap^37^. The relationship between genetic and physical distances was first visualized to remove outlier markers resulting from scaffold misplacement within pseudo-chromosomes. We then used the Loess interpolation method which estimates regional recombination rates by locally adjusting a 2nd degree polynomial curve, setting the span parameter value to 0.25.

The cumulative recombination frequency (RFm) along LGs was used to infer the chromosome type as previously described^38^. Briefly, this approach computes the number of recombination events from both ends of each LG using each terminal marker as a reference starting point. For acrocentric chromosomes, RFm will be 0.0 at the terminal reference marker and increases linearly towards a value of 0.5 at the opposite end. For metacentric chromosomes, plots of RFm are expected to reach a plateau along the chromosome arm opposite to the terminal reference marker.

A highly detailed analysis of synteny across flatfish is beyond the scope of this study, but a macrosynteny analysis was carried out to identify chromosomal rearrangements in flatfish. The pseudo-chromosomes of *S.* *senegalensis* (family Soleidae) were compared with assembled chromosome sequences of turbot (*S.* *maximus*, Scophthalmidae; GenBank assembly accession: GCA_013347765.1), Japanese flounder (*Paralichthys* *olivaceus*, Paralichthyidae; Acc.N. GCA_001904815.1) and tongue sole (*C.* *semilaevis*, Cynoglossidae; Acc.N. GCF_000523025.1) using D-Genies^39^.

**References**

1 Wick, R. R., Judd, L. M., Gorrie, C. L. & Holt, K. E. Completing bacterial genome assemblies with multiplex MinION sequencing. *Microb Genom* **3**, e000132, doi:<https://doi.org/10.1099/mgen.0.000132> (2017).

2 Zimin, A. V. *et al.* Hybrid assembly of the large and highly repetitive genome of *Aegilops tauschii*, a progenitor of bread wheat, with the MaSuRCA mega-reads algorithm. *Genome Res.* **27**, 787-792, doi:<https://doi.org/10.1101/gr.213405.116> (2017).

3 Zimin, A. V. *et al.* The MaSuRCA genome assembler. *Bioinformatics* **29**, 2669-2677, doi:<https://doi.org/10.1093/bioinformatics/btt476> (2013).

4 Simao, F. A., Waterhouse, R. M., Ioannidis, P., Kriventseva, E. V. & Zdobnov, E. M. BUSCO: assessing genome assembly and annotation completeness with single-copy orthologs. *Bioinformatics* **31**, 3210-3212, doi:<https://doi.org/10.1093/bioinformatics/btv351> (2015).

5 Waterhouse, R. M. *et al.* BUSCO Applications from Quality Assessments to Gene Prediction and Phylogenomics. *Mol Biol Evol* **35**, 543-548, doi:<https://doi.org/10.1093/molbev/msx319> (2018).

6 Walker, B. J. *et al.* Pilon: an integrated tool for comprehensive microbial variant detection and genome assembly improvement. *PloS one* **9**, e112963, doi:<https://10.1371/journal.pone.0112963> (2014).

7 Brown, J. K. *et al.* Mapping the sex determination locus in the hapuku (*Polyprion* *oxygeneios*) using ddRAD sequencing. *BMC genomics* **17**, 448, doi:<https://doi.org/10.1186/s12864-016-2773-4> (2016).

8 Peterson, B. K., Weber, J. N., Kay, E. H., Fisher, H. S. & Hoekstra, H. E. Double digest RADseq: an inexpensive method for de novo SNP discovery and genotyping in model and non-model species. *PloS one* **7**, e37135, doi:<https://doi.org/10.1371/journal.pone.0037135> (2012).

9 Rochette, N. C. & Catchen, J. M. Deriving genotypes from RAD-seq short-read data using Stacks. *Nat Protoc* **12**, 2640-2659, doi:<https://doi.org/10.1038/nprot.2017.123> (2017).

10 Purcell, S. *et al.* PLINK: a tool set for whole-genome association and population-based linkage analyses. *Am J Hum Genet* **81**, 559-575, doi:<https://doi.org/10.1086/519795> (2007).

11 Rastas, P. Lep-MAP3: robust linkage mapping even for low-coverage whole genome sequencing data. *Bioinformatics* **33**, 3726-3732, doi:<https://doi.org/10.1093/bioinformatics/btx494> (2017).

12 Ouellette, L. A., Reid, R. W., Blanchard, S. G. & Brouwer, C. R. LinkageMapView-rendering high-resolution linkage and QTL maps. *Bioinformatics* **34**, 306-307, doi:<https://doi.org/10.1093/bioinformatics/btx576> (2018).

13 Rastas, P. Lep-Anchor: automated construction of linkage map anchored haploid genomes. *Bioinformatics* **36**, 2359-2364, doi:<https://doi.org/10.1093/bioinformatics/btz978> (2020).

14 Morgulis, A., Gertz, E. M., Schaffer, A. A. & Agarwala, R. WindowMasker: window-based masker for sequenced genomes. *Bioinformatics* **22**, 134-141, doi:<https://doi.org/10.1093/bioinformatics/bti774> (2006).

15 Huang, S., Kang, M. & Xu, A. HaploMerger2: rebuilding both haploid sub-assemblies from high-heterozygosity diploid genome assembly. *Bioinformatics* **33**, 2577-2579, doi:<https://doi.org/10.1093/bioinformatics/btx220> (2017).

16 Guerrero-Cózar, I. *et al.* Development of whole-genome multiplex assays and construction of an integrated genetic map using SSR markers in Senegalese sole. *Sci. Reports* **10**, 21905, doi:<https://doi.org/10.1038/s41598-020-78397-w> (2020).

17 Claros, M. G., Seoane, P. & Manchado, M. Sequences and annotations of a provisional genome draft of a Senegalese sole female. figshare <https://doi.org/10.6084/m9.figshare.12472100.v1>. (2020).

18 Altschul, S. F., Gish, W., Miller, W., Myers, E. W. & Lipman, D. J. Basic local alignment search tool. *J Mol Biol* **215**, 403-410, doi:<https://10.1016/S0022-2836(05)80360-2> (1990).

19 Dobin, A. *et al.* STAR: ultrafast universal RNA-seq aligner. *Bioinformatics* **29**, 15-21, doi:<https://10.1093/bioinformatics/bts635> (2013).

20 Pertea, M. *et al.* StringTie enables improved reconstruction of a transcriptome from RNA-seq reads. *Nat Biotechnol* **33**, 290-295, doi:<https://10.1038/nbt.3122> (2015).

21 Niknafs, Y. S., Pandian, B., Iyer, H. K., Chinnaiyan, A. M. & Iyer, M. K. TACO produces robust multisample transcriptome assemblies from RNA-seq. *Nat Methods* **14**, 68-70, doi:<https://10.1038/nmeth.4078> (2017).

22 Mapleson, D., Venturini, L., Kaithakottil, G. & Swarbreck, D. Efficient and accurate detection of splice junctions from RNA-seq with Portcullis. *GigaScience* **7**, doi:<https://10.1093/gigascience/giy131> (2018).

23 Haas, B. J. *et al.* Automated eukaryotic gene structure annotation using EVidenceModeler and the Program to Assemble Spliced Alignments. *Genome biology* **9**, R7, doi:<https://10.1186/gb-2008-9-1-r7> (2008).

24 Iwata, H. & Gotoh, O. Benchmarking spliced alignment programs including Spaln2, an extended version of Spaln that incorporates additional species-specific features. *Nucleic acids research* **40**, e161, doi:<https://10.1093/nar/gks708> (2012).

25 Parra, G., Blanco, E. & Guigo, R. GeneID in Drosophila. *Genome research* **10**, 511-515, doi:<https://10.1101/gr.10.4.511> (2000).

26 Stanke, M., Schoffmann, O., Morgenstern, B. & Waack, S. Gene prediction in eukaryotes with a generalized hidden Markov model that uses hints from external sources. *BMC bioinformatics* **7**, 62, doi:<https://doi.org/10.1186/1471-2105-7-62> (2006).

27 Lomsadze, A., Burns, P. D. & Borodovsky, M. Integration of mapped RNA-Seq reads into automatic training of eukaryotic gene finding algorithm. *Nucleic acids research* **42**, e119, doi:<https://10.1093/nar/gku557> (2014).

28 Conesa, A. *et al.* Blast2GO: a universal tool for annotation, visualization and analysis in functional genomics research. *Bioinformatics* **21**, 3674-3676, doi:<https://doi.org/10.1093/bioinformatics/bti610> (2005).

29 Jones, P. *et al.* InterProScan 5: genome-scale protein function classification. *Bioinformatics* **30**, 1236-1240, doi:<https://10.1093/bioinformatics/btu031> (2014).

30 Paten, B. *et al.* Cactus: Algorithms for genome multiple sequence alignment. *Genome research* **21**, 1512-1528, doi:<https://10.1101/gr.123356.111> (2011).

31 Figueras, A. *et al.* Whole genome sequencing of turbot (*Scophthalmus* *maximus*; Pleuronectiformes): a fish adapted to demersal life. *DNA Res* **23**, 181-192, doi:<https://doi.org/10.1093/dnares/dsw007> (2016).

32 Fiddes, I. T. *et al.* Comparative Annotation Toolkit (CAT)-simultaneous clade and personal genome annotation. *Genome research* **28**, 1029-1038, doi:<https://10.1101/gr.233460.117> (2018).

33 Cui, X., Lu, Z., Wang, S., Jing-Yan Wang, J. & Gao, X. CMsearch: simultaneous exploration of protein sequence space and structure space improves not only protein homology detection but also protein structure prediction. *Bioinformatics* **32**, i332-i340, doi:<https://10.1093/bioinformatics/btw271> (2016).

34 Nawrocki, E. P. & Eddy, S. R. Infernal 1.1: 100-fold faster RNA homology searches. *Bioinformatics* **29**, 2933-2935, doi:<https://10.1093/bioinformatics/btt509> (2013).

35 Nawrocki, E. P. *et al.* Rfam 12.0: updates to the RNA families database. *Nucleic acids research* **43**, D130-137, doi:<https://10.1093/nar/gku1063> (2015).

36 Chan, P. P. & Lowe, T. M. tRNAscan-SE: Searching for tRNA Genes in Genomic Sequences. *Methods Mol Biol* **1962**, 1-14, doi:10.1007/978-1-4939-9173-0_1 (2019).

37 Rezvoy, C., Charif, D., Gueguen, L. & Marais, G. A. MareyMap: an R-based tool with graphical interface for estimating recombination rates. *Bioinformatics* **23**, 2188-2189, doi:<https://doi.org/10.1093/bioinformatics/btm315> (2007).

38 Limborg, M. T., McKinney, G. J., Seeb, L. W. & Seeb, J. E. Recombination patterns reveal information about centromere location on linkage maps. *Molecular ecology resources* **16**, 655-661, doi:<https://doi.org/10.1111/1755-0998.12484> (2016).

39 Cabanettes, F. & Klopp, C. D-GENIES: dot plot large genomes in an interactive, efficient and simple way. *PeerJ* **6**, e4958, doi:<https://doi.org/10.7717/peerj.4958> (2018).
